# From grief, guilt pain and stigma to hope and pride – a systematic review and meta-analysis of mixed-method research of the psychosocial impact of stillbirth

**DOI:** 10.1186/s12884-016-0800-8

**Published:** 2016-01-19

**Authors:** Christy Burden, Stephanie Bradley, Claire Storey, Alison Ellis, Alexander E. P. Heazell, Soo Downe, Joanne Cacciatore, Dimitrios Siassakos

**Affiliations:** NIHR Clinical Lecturer in Obstetrics and Gynaecology, University of Bristol, School of Social & Community Medicine, Obstetrics and Gynaecology, Southmead Hospital, Westbury on Trym, Bristol, BS10 5NB UK; Primary Care Librarian, Southmead Hospital, Westbury on Trym, Bristol, BS10 5NB UK; International Stillbirth Alliance, London, UKᅟ; Specialist Registrar, Obstetrics & Gynaecology, Southmead Hospital, Westbury on Trym, Bristol, BS10 5NB UK; Maternal & Fetal Health Research Centre, Institute of Human Development, University of Manchester, Manchester, UK; St Mary’s Hospital, Central Manchester University Hospitals NHS Foundation Trust, Manchester Academic Health Science Centre, Manchester, M13 9WL UK; School of Health, University of Central Lancashire, Brook Building, Lancashire, PR1 2HE UK; School of Social Work, Arizona State University, Phoenix, AZ USA; Obstetrics and Gynaecology, University of Bristol, School of Social & Community Medicine, Obstetrics and Gynaecology, Southmead Hospital, Westbury on Trym, Bristol, BS10 5NB UK

**Keywords:** Stillbirth, Bereavement, Psychosocial impact, Parents’ experiences

## Abstract

**Background:**

Despite improvements in maternity healthcare services over the last few decades, more than 2.7 million babies worldwide are stillborn each year. The global health agenda is silent about stillbirth, perhaps, in part, because its wider impact has not been systematically analysed or understood before now across the world. Our study aimed to systematically review, evaluate and summarise the current evidence regarding the psychosocial impact of stillbirth to parents and their families, with the aim of improving guidance in bereavement care worldwide.

**Methods:**

Systematic review and meta-summary (quantitative aggregation of qualitative findings) of quantitative, qualitative, and mixed-methods studies. All languages and countries were included.

**Results:**

Two thousand, six hundred and nineteen abstracts were identified; 144 studies were included. Frequency effect sizes (FES %) were calculated for each theme, as a measure of their prevalence in the literature.

Themes ranged from negative psychological symptoms post bereavement (77 · 1) and in subsequent pregnancies (27 · 1), to disenfranchised grief (31 · 2), and incongruent grief (28 · 5), There was also impact on siblings (23 · 6) and on the wider family (2 · 8).

They included mixed-feelings about decisions made when the baby died (12 · 5), avoidance of memories (13 · 2), anxiety over other children (7 · 6), chronic pain and fatigue (6 · 9), and a different approach to the use of healthcare services (6 · 9).

Some themes were particularly prominent in studies of fathers; grief suppression (avoidance)(18 · 1), employment difficulties, financial debt (5 · 6), and increased substance use (4 · 2). Others found in studies specific to mothers included altered body image (3 · 5) and impact on quality of life (2 · 1). Counter-intuitively, Some themes had mixed connotations. These included parental pride in the baby (5 · 6), motivation for engagement in healthcare improvement (4 · 2) and changed approaches to life and death, self-esteem, and own identity (25 · 7).

In studies from low/middle income countries, stigmatisation (13 · 2) and pressure to prioritise or delay conception (9) were especially prevalent.

**Conclusion:**

Experiencing the birth of a stillborn child is a life-changing event. The focus of the consequences may vary with parent gender and country. Stillbirth can have devastating psychological, physical and social costs, with ongoing effects on interpersonal relationships and subsequently born children. However, parents who experience the tragedy of stillbirth can develop resilience and new life-skills and capacities. Future research should focus on developing interventions that may reduce the psychosocial cost of stillbirth.

**Electronic supplementary material:**

The online version of this article (doi:10.1186/s12884-016-0800-8) contains supplementary material, which is available to authorized users.

## Background

Across the globe in 2015, 2.7 million babies were stillborn [1]. Rates of stillbirth vary from 2 · 0 per 1000 total births in Finland, 4.6 per 1000 in the UK to more than 40 per 1000 total births in Nigeria, Ethiopia and Pakistan [2–4]. Despite underreporting, 98 % of stillbirths occur in low and middle-income countries (LMIC), and 67 % occur in rural families [3, 4]. Furthermore, stillbirth is still not acknowledged as a serious public health issue on the global health agenda [5].

Stillbirth can be a devastating life event for women and their partners. Although it has been shown to cause prolonged grief that is comparable to any death of a child, the grief that results after a stillbirth or neonatal death has been described as complex and unique [6] at least in part because of a lack of acceptance or legitimisation of the grieving process by society. Moreover, as the majority women conceive within a year of the loss [7], negative psychological effects of the loss may continue into subsequent pregnancies, despite the birth of a healthy child [8].

However, the exact extent of the wider impact on families, society, government and healthcare services remains unknown and is likely under-estimated. No previous study has systematically analysed the short and long term psychological and social effects associated with stillbirth in high-income countries (HICs) and LMICs. The aim of this systematic review was to assess the current available evidence on the impact of stillbirth, with the aim of improving awareness of the psychosocial impact of stillbirth and informing the development of international guidance on care of bereaved parents and their families

## Methods

### Study design

We conducted a systematic review and meta-summary of published studies that evaluated the experiences of stillbirth for parents and the immediate family. Only previously published studies were used, so there was no requirement for ethical review.

### Data information sources

**Fig. 1:**
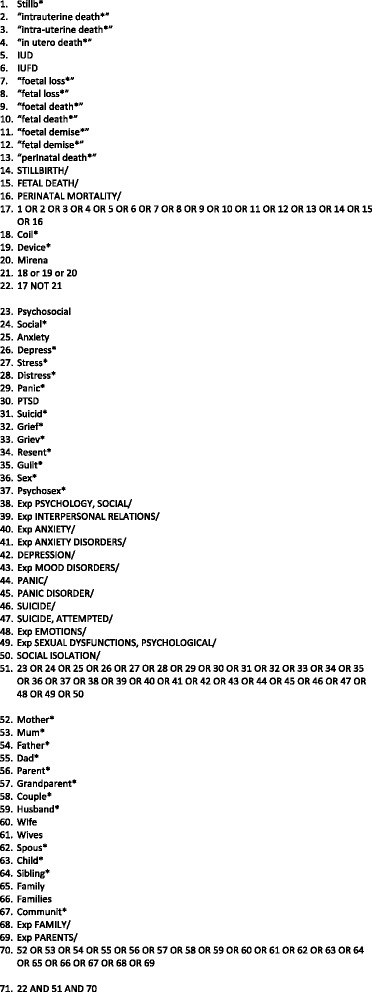
Search Strategy: MeSH Headings for CINHAL. **subject headings varied between databases but covered the same topic/concept

Search terms (Fig. 1) were formulated based on an interpretation of the population/problem of interest, intervention and context (or PICO) and SPIDER (Sample, Phenomenon of Interest, Design, Evaluation, Research type) frameworks [9]. Using these frameworks and following the PRISMA guidelines, we performed a literature search using Medline, PUBMED, Embase, Scopus, Amed, BNI, CINAHL and PsycINFO, together with conference abstracts from Royal College of Obstetricians & Gynaecologists (RCOG) and International Stillbirth Alliance (ISA) from January 2000 to February 2015.

### Eligibility criteria

Qualitative, quantitative and mixed methods studies were included if they assessed at least one psychosocial impact/experience of stillbirth on parents, grandparents, siblings or future children (including a surviving multiple). All languages and countries (HIC and LMIC) [10] were included. We excluded studies prior to January 2000, studies assessing impact on healthcare professionals, reviews, dissertations, and books unless they included original data. Studies assessing impact exclusively after lethal fetal diagnosis, termination of pregnancy, miscarriage or neonatal death were excluded. It was decided to include studies after January 2000 to focus on up-to-date evidence.

### Study selection

Combining search results provided an initial screen. Four investigators (CB, SB, DS and CS), excluded studies through screening abstracts. Reasons for exclusion were: a) duplicates, b) topic not relevant to stillbirth, c) impact on healthcare professionals not parents, d) review articles, e) year of publication, or f) dissertations. Reference lists were scanned for additional studies. This generated a list of potential full text articles, which were obtained and assessed independently by two investigators (CB and SB). Further articles were excluded using the same criteria, and also if i) no findings found in data, and j) articles requiring complex translation, k) article unavailable. Disagreements on whether to include or exclude articles were discussed between the wider research team to reach consensus. We sought any unclear or missing information by contacting the authors of the individual studies.

### Data extraction

The findings were extracted from the final articles by two investigators (CB & SB). Meta-analysis and extraction of data was based on the ‘meta-summary’ approach, a quantitatively oriented aggregation of qualitative findings developed by Sandelowski et al. [11]. A core investigator group (CB, SB, CS, AE, DS) summarized the findings in thematic sentences, and a further investigator with psychology qualifications (JC) revised the categorical wording.

### Summary measures & synthesis of results

To assess the relative importance of the themes (thematic sentences) frequency effect sizes (FES) were calculated. FES were calculated by taking the number of articles containing a theme (minus any articles derived from a common parent study and representing a duplication of the same finding) and dividing this number by the total number of articles [11]. The higher the FES, the greater the relative magnitude of the associated abstracted finding [11]. In a sub group analysis the FES were calculated by; the country of origin of the article (HIC or LMIC), and by participants (mothers or fathers). To assess the degree to which articles contributed to the final set of abstracted themes, we calculated the intensity effect size (IES) of each article. The IES was derived for each included article by dividing the number of themes contained in the articles by the total number of themes across all reports [11].

## Results

### Study selection

**Fig. 2:**
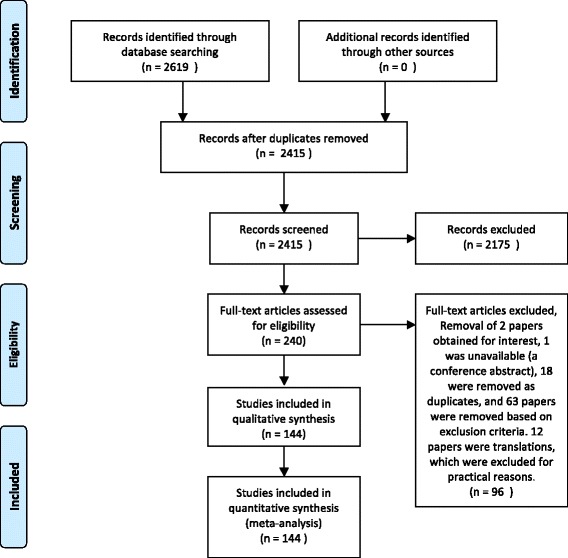
Flow diagram of search methodology

Two thousand, six hundred and nineteen abstracts were identified. After duplicate removal and eligibility screening, 240 studies were selected for full text review, and 144 articles were included in the final analysis (Fig. 2).

### Findings

**Fig. 3:**
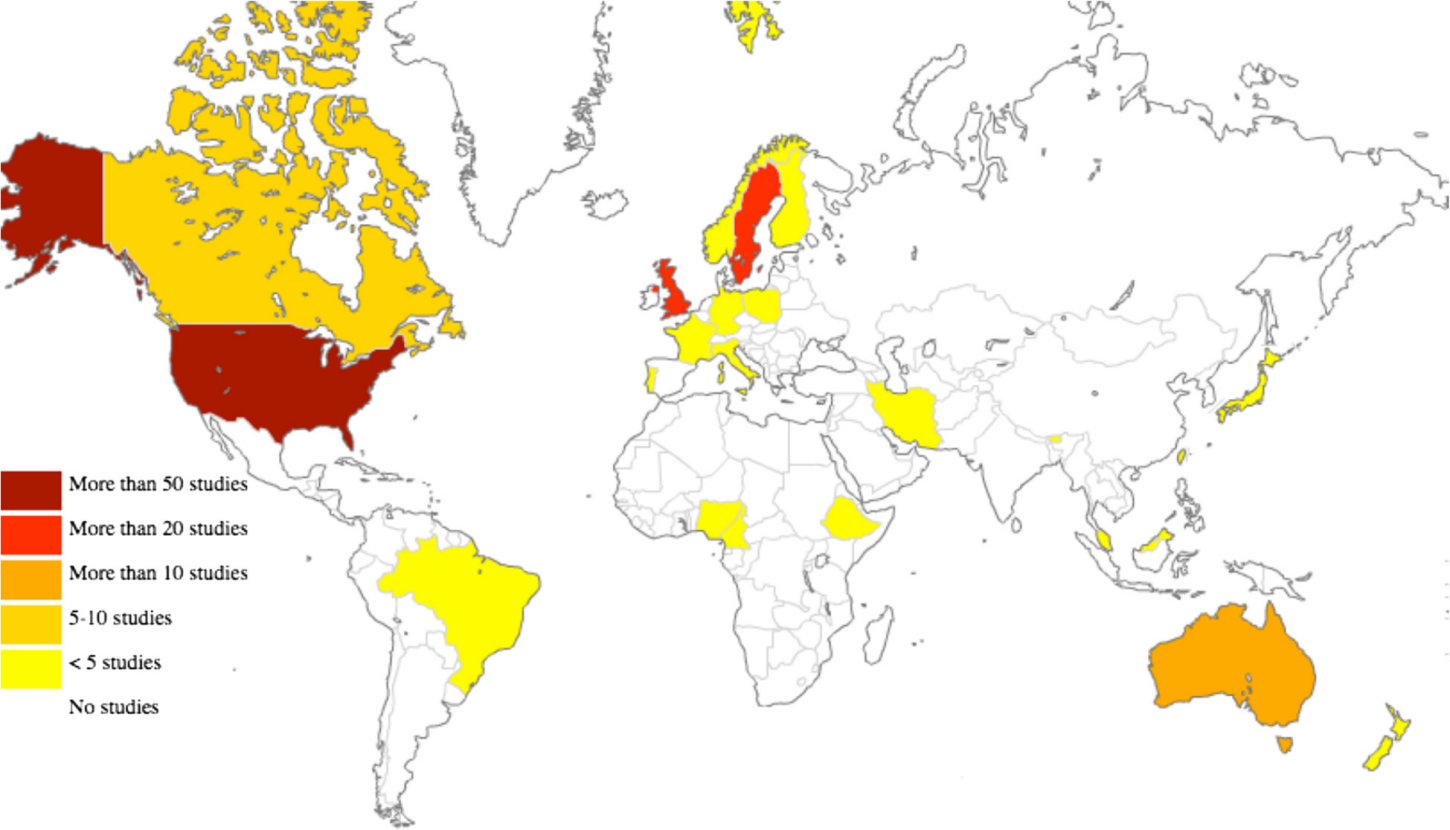
Location of studies included in the meta-analysis of the psychosocial impact of stillbirth

**Fig. 4:**
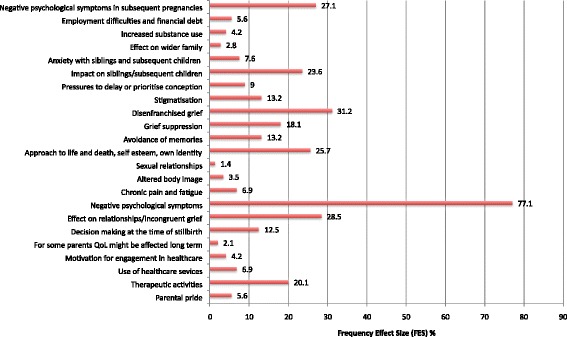
Total frequency effect sizes—FES % for all thematic sentences

Articles were published over a 15-year period from January 2000 to February 2015, reporting studies conducted in 25 countries worldwide (Fig. 3). 129 studies were from HIC, 9 from UMIC, 3 from LMIC, and 3 from LIC (Additional files 1 and 2). Overall 1110 individual findings were extracted from the studies and 23 themes with thematic sentences were identified and used for calculation of FES (Additional file 2: Table S1; Fig. 4) and IES (Additional file 1).

### Themes

*Stillbirth is associated with a number of emotional, depressive and other negative psychological symptoms.* Bereaved parents had significantly higher rates of psychological and emotional disorders including; depression (both self reported and clinical), general anxiety disorder, social phobia, agoraphobia, anger, negative cognitive appraisals such as a sense of failure and long-term guilt and other post traumatic stress disorder (PTSD) symptoms, and suicidal ideation. Some parents were shown to experience strong feelings of social isolation and disconnection from their social environment. Mental health issues, in some instances, arose decades after the loss. Factors described as influencing these responses to stillbirth are described in Table 1.

**Table 1:** Influencing factors for depression & negative psychological symptoms after stillbirth

Influencing factors	
Negative	
Previous perinatal loss	
Complications in index pregnancy	
Poor support from partner	
Increased time from index pregnancy	
Increased maternal age	
Psychological problems pre-existing	
Gender (female)	
Grief suppression	
Death of partner (symptoms may re-emerge)	
Not conceiving in future	
Absence of living children	
Personality traits	
Not seeing/holding baby/saying goodbye	
Lack of maternal pride	
Single/divorced or widowed parent	
Poor family support/social network	
Increased number of pregnancy losses	
Prolonged grief	
History of sexual or physical abuse	
Attending ultrasound in index pregnancy (males)	
Lack of male support for fathers	
Ambiguity of burial arrangements	
One partner being ignored by HCP	
Unemployment	
History of infertility	
Multiple pregnancy losses	
Seeking cause of loss/blame	
Delay in IOL after loss	
Insensitive treatment by HCP	
Poor ego strength (males)	
History of stressful life events	
Positive	
Treatment/counselling/professional support	

*HCP* Health care professional

*IOL* Induction of labour

*Stillbirth may lead to avoidance of activities* where parents may come into contact with babies or anything that reminded them of their own losses, creating voluntary social isolation.

*Disenfranchised grief; parental grief following stillbirth may not legitimised by health professionals, family and society.* Parents felt isolated, noting their identity as parents was not recognised by society; they were a parent, but without a child. Fathers especially reported that they felt marginalised and unacknowledged as a grieving parent. Parents recounted experiences suggesting that relationships with others had changed irrevocably. Many parents found if hurtful when their baby was referred to as less than a person, as something replaceable and not to be remembered as part of their family. Many parents indicated that mourning the death of a newborn was taboo and not culturally acceptable.

*Incongruent grieving styles; stillbirth may have an impact on relationships, for example through different grief reactions.* Divorce and relationship difficulties after stillbirth were frequently reported. The different grieving patterns or ‘incongruent grief’ of mothers and fathers were often cited as reason for these difficulties. For some couples this led to disputes, infidelity and, at times, physical violence. In contrast, some couples stated that they became closer after the loss and now had a ‘special unifying bond’. *Some couples reported experiencing conflicting emotional reactions to sexual relationships.* Women, more frequently than men reported guilt and disturbing images, thoughts and feelings that interfered with sex.

*Parents may experience external or internal pressures to prioritise or delay conception.* Some women described feeling pressured to prove their reproductive capabilities as soon as possible and that the desire to have a newborn to nurture could be overwhelming. Others wanted to delay another pregnancy due to the concern over recurrence of a stillbirth. Equally, some parents felt that they could not contemplate ‘replacing’ that baby and needed to wait until they felt ready. Others described external pressures from well meaning others who likely believed another baby would replace the stillborn baby.

*In subsequent pregnancies, some parents may feel isolated and outside the boundaries of normality, and they experience a number of emotional responses* including *depressive and other psychological symptoms.* Many parents reported that they were unable to feel the normal excitement, anticipation and bonding during pregnancy, and were unable to participate in antenatal classes for a number of reasons including fear of the negative impact on other parents, envy of the joys of others, and a fear of being shunned by others in the group. Parents experienced a continuum of emotions from relief to anxiety, from hopeful optimism to panic and anxiety attacks, isolation and lack of normality. Parents also described the contrast of life and death, as they feared losing their current baby and found it difficult to separate the concepts of life and death. Both mothers and fathers reported feeling anxious, and fathers wanted to be more involved with the obstetric care in the subsequent pregnancy.

Stillbirth was shown to have an impact on the wider family, including grandparents. *Stillbirth can also have an adverse impact on siblings and complicate attachment for parents*, *including the surviving twin, and subsequent children.* These effects appeared to be long lasting, and could impact children’s long-term mental and physical health.

Some parents reported feeling torn between managing their own grief and parenting siblings, whilst others found comfort at the time of grief from existing siblings. Support from relatives, could mean that siblings were also sometimes physically separated from their parents. This could lead to them being temporarily distanced emotionally from their parents during the grief process.

A factor that contributed to complicated attachment for a few parents was a sibling’s resemblance to the stillborn baby. This was reported with both subsequent children and loss in a multiple pregnancy. Many parents reported feeling emotionally guarded about their living children for fear of losing them and going through repeated grief. The long-term impact on siblings, surviving twins and subsequent children also included survivor guilt as they felt that they had to live their life for two people.

*After stillbirth some parents may alter their activities as a coping strategy including; seeking therapeutic isolation (needing time to themselves), increased or decreased religious activity, increased or decreased sexual activity, and increased engagement with health promoting activities, work and social media. This may all continue into subsequent pregnancies.*

Exercise was a form of therapy for some parents and helped them to deal with their emotions and improved symptoms of depression i.e. gave more energy and enthusiasm, it was seen as a means of coping. Several parents felt that by practicing religious activities they were able to reduce the pain they suffered and make their mind more accepting of the situation. For others it did not, with a small minority admitting they felt God may have been responsible for the stillbirth. Some parents acknowledged that sexual activity could serve to reduce tension and described it as therapeutic; this was more commonly reported in men. Others avoided sexual activity due to lack of interest. Lastly, a number of parents felt social media provided them with space to talk openly about their loss and its implications. This was valuable to do because often they did not feel safe having these conversations elsewhere.

**Fig. 5:**
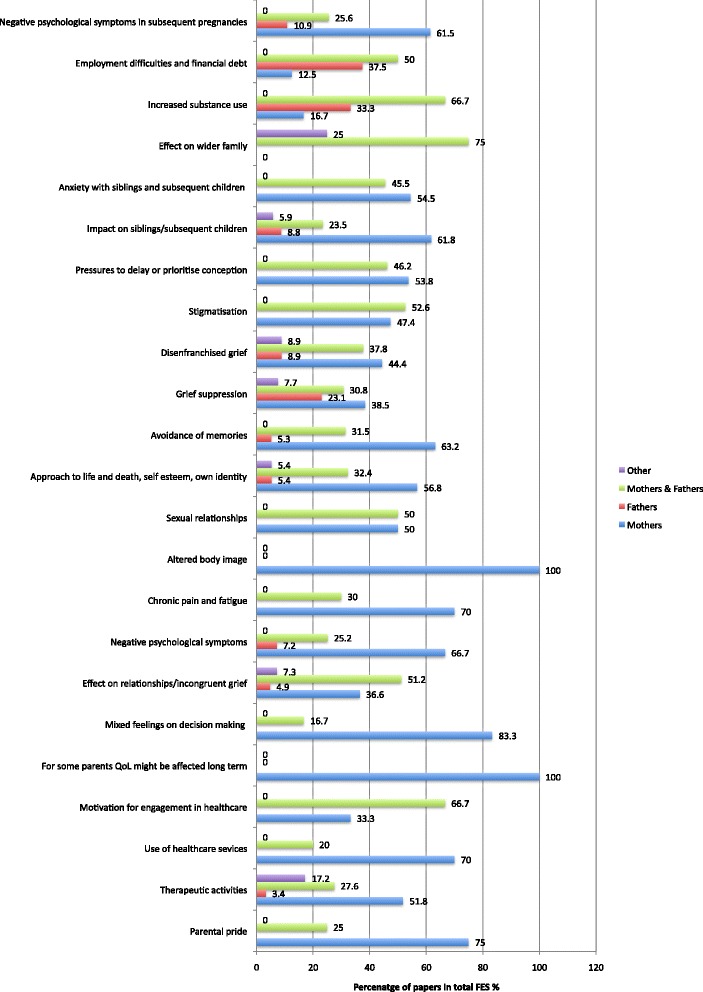
Subanalysis of frequency size effects by parents gender

*Some parents felt the need to suppress outward grief, including during subsequent pregnancy*. For fathers, especially (Fig. 5) those who perceived their social role as needing to provide emotional support for their partner and family, the burden of keeping feelings to themselves may lead to grief suppression, potentially increasing the risk of chronic psychological issues. Many mothers, most notably in LMICs, also often dealt with their grief privately and alone. Suppression of grief for both parents was reported to lead to relationship difficulties within the couple and also the wider family unit.

**Fig. 6:**
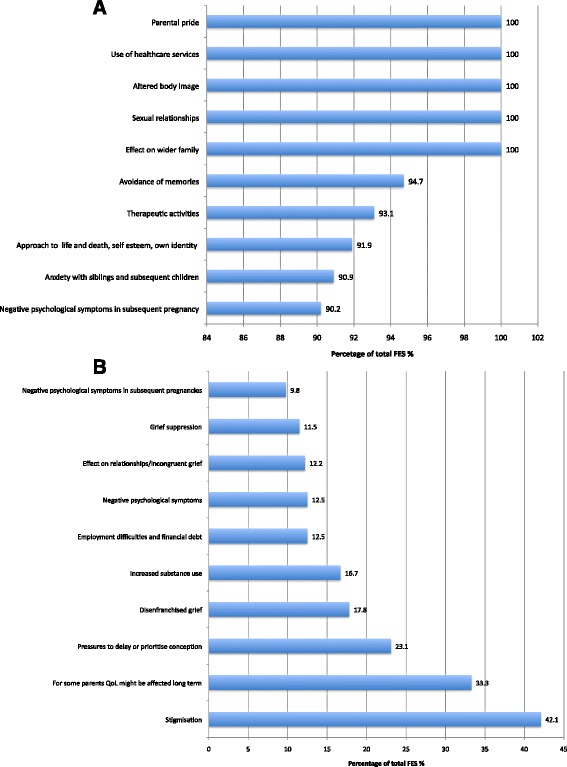
**a** Subanalysis of frequency size effect socioeconomic status of country—Five themes with highest percentage of total frequency effect sizes in high income countries. **b**—Subanalysis of frequency size effect socioeconomic status of country—Five themes with highest percentage of total frequency effect sizes in low/middle income countries

*Women reported stigmatisation, rejection, and spousal abuse from their partner, family and society.* This was most notably reported in the majority of LMIC (Table 1; Fig. 6a and b). Women were frequently blamed for the death of their babies and some were thought to be under the spell of evil spirits or have tried to procure an abortion. There were reports of women being avoided, sent back to work immediately after giving birth, being divorced by their partner, suffering physical abuse, and even being forced out of their villages, thus leaving them destitute [12].

*Parents may have mixed feelings regarding the decisions they made*. Many parents reported conflicting emotions upon later re-evaluating the decisions they made when their baby was born. These included seeing and spending time with their stillborn baby, memory-making rituals, and whether or not to have a post mortem evaluation or autopsy. The majority of parents expressed regrets about their decision not to hold or spend time with their stillborn baby. Many parents voiced a clear sense of frustration and injustice at having their decisions influenced by insufficient or inaccurate information provided by professionals, as they were scared or unsure what they were ‘allowed to do’, which later led to regret.

*Bereaved parents may become hypervigilant with siblings, their subsequent children, and anxious about other people's children.* Parents shared stories of feeling anxious and out of control, especially when faced with ‘normal’ or ‘common’ childhood events or illness. These events led them to feel intense fear and concern that they may lose another child.

*Chronic pain and fatigue were also shown to follow stillbirth for some parents. It was also reported that bereaved parents may increase or decrease their use of health care services.* Some parents were reported to be more likely to have health challenges in subsequent pregnancies and about their subsequent children. This resulted in increased phone calls and visits to health care providers [13]. However in subsequent pregnancies some parents reported that they withdrew from healthcare as a protective mechanism.

*The potential impact of stillbirth included employment difficulties and financial debt—*this occurred especially in LMIC because of hospital bills and funeral expenses, which often further increased family relationship tensions [14]. This impact was commonly reported by fathers (Fig. 5). However, mothers also reported being more physically and mentally exhausted from work or more likely to be on sick leave than non-bereaved mothers. Some employed parents also noted difficulty concentrating or emotional breakdowns at work. One mother perceived feeling unwanted back when she returned to work.

*Increased substance use has been reported for some parents.* This was another finding more commonly reported in fathers (Fig. 5). Only one study reported increased alcohol and substance use in mothers.

*Women may develop a complex emotional response to body image*. Many mothers blamed themselves for the baby’s death, citing their “body’s failure”. Women were embarrassed and guilty of their post pregnant bodies as they did not have a baby. Conversely some women wanted to keep their bodies in a pregnant shape to stay connected to the baby. A number of women linked the grief to their body, both through physical pain and by developing an image of their body as unattractive and ugly, which also decreased sexual activity and pleasure.

*It was also reported that Quality of Life (QoL) might be affected for parents in the long-term,* although this was not the case in all studies, with some research showing no evidence of impact on QoL.

*Stillbirth may change parents’ approach to life and death, self-esteem, identity, and sense of control in subsequent pregnancy, parenthood and childrearing. As a result of stillbirth, some parents* felt themselves to be more caring, thoughtful and compassionate, less materialistic and less likely to “take anything for granted”, but several women stated that after stillbirth they did not feel “whole”, that something had changed in their identity as a woman. Others reported increased or decreased fear of death after stillbirth. Many women perceived themselves as failures at the role of mother, wife, daughter and daughter-in-law. Fathers’ responses to stillbirth often corresponded with feelings of failure in the role of provider and protector.

*Some parents described parental pride after the birth of their stillborn baby.* Some studies described that parents felt a strong sentiment of parental pride, and that meeting, seeing and holding their child strengthened their feelings as a parent, and that this feeling, at least temporarily, took over from the shock of the death. Some women felt that they were able to endure the experience of labour by looking forward to seeing their baby, [15] and a number of parents found solace in identifying family traits.

*Stillbirth can motivate parents to engage with healthcare improvement and public awareness.* Some parents found taking part in research projects, providing peer support, lobbying for stillbirth and working with hospitals to improve service provision and care beneficial and perceived it as a way in which they could help to improve care for the future couples and families.

## Discussion

### Main findings

The findings from this review have demonstrated that stillbirth often has profound long-term detrimental psychological, physical, social, and financial impacts on parents, and the immediate family. It can influence their relationships, and its effects can extend into subsequent pregnancies and parenthood. The effects of stillbirth are likely to be associated with significantly increased costs to healthcare services and society. These costs have been related to negative psychological symptoms, reduced social functioning and family unit breakdown, reduced financial status and employment, and increased healthcare utilization in subsequent healthy pregnancies.

The majority of large frequency effects are seen in themes that demonstrate the devastating impact of stillbirth. However, unlike other evidence in this general area, this review also identified some areas of engagement and personal growth that likely evolve through processing experiences with effective provision of support and care.

### Strengths and limitations

The main strength of this comprehensive review is that we included studies of varied methodology, both qualitative, mixed method and quantitative, and did not exclude non-English language articles. It included studies from 25 different countries, with participants from different cultures and religions, increasing the external validity of our findings. We excluded studies assessing the impact of miscarriages and neonatal death. Despite the inclusive design of the review the large majority of studies were, however, undertaken in HIC compared to LMIC. We therefore separated the themes by country to enable conclusions about their relevant importance in different settings.

The main limitation is the variable quality of included studies. This inclusive approach is advised in the ‘Metasummary’ method because it is considered that all studies can yield useful information that arbitrary quality systems risk excluding. In reporting intensity effect sizes we have allowed readers to ascertain if any findings were obtained from largely ‘weaker’ studies, which articles contributed most of the findings with the largest FES, and which articles contributed findings that no other articles contained [11].

### Interpretation of results

A previous review has assessed the impact of stillbirth and neonatal death on subsequent pregnancy, but was limited to studies in HIC and parents alone, not the wider family [16]. A further comprehensive systematic review conducted by the Joanna Briggs Institute for the Stillbirth Foundation Australia aimed to identify care and support strategies for families to improve their psychological wellbeing after stillbirth. Although this review focussed on the available evidence surrounding the experiences of care provided to families who had a stillbirth, similar key themes in parents’ experiences were identified including; conflict with decision-making at the time of stillbirth, the need to acknowledge parenthood and the long-term impact of stillbirth in subsequent pregnancies [17]. Our review demonstrated some controversy with decision-making at the time of stillbirth, with many parents agonising over both the decisions they made and the decisions they did not make. To have the opportunity to say goodbye, to see their baby, make memories or have a postmortem evaluation brought about a sense of finality that for many parents was perceived as contributing to the healing process. Some studies have demonstrated these opportunities decreased anxiety, reduced physical symptoms and sleep disorders and allowed parents to cope better with their grief [18–21].

Findings surrounding the use of therapeutic activities after pregnancy loss, although give us information on how some parents may react and cope after stillbirth, demonstrate the complexity of the subject. For example, increased sexual activity is intrinsically entwined with other themes such as, body image and sexual relationships, emotional wellbeing and depression, guilt at doing something pleasurable, physical pain and desire to conceive or not conceive again (which can be different between couples). Caution, must therefore be taken in interpreting the findings, and it should be acknowledged that individual families will respond differently and that their care will need to be individualised to reflect these differences.

Many of the themes in this review are similar between HICs and LMICs. Stillbirth was widely believed by society to be a natural selection of babies never meant to live. Stigmatisation has been especially reported in LMICs [22]. The beliefs in the mother’s sins and evil spirits as causes of stillbirth appear rife in some LMICs. As a result of cultural beliefs and societal pressures, parents in LMICs are often reluctant to talk about or see the stillborn infant, preventing them from accessing rituals that may be helpful including seeing, baptising, and naming the stillborn baby as a means of acknowledging his or her existence. Moreover, because of this fear of stigmatization, stillbirth in LMICs is likely underreported [23]. Future research is therefore needed as a priority across countries of different religions, cultures and economic status to establish the true extent and cost of the impact of stillbirth worldwide. Furthermore, addressing stigma and taboo should be a priority, particularly in LMICs. This study suggests that much of the stigma comes from communities and societies. Therefore, messages to counteract stigma will need to be developed and distributed in conjunction with relevant community stakeholders and leaders.

Grief, and sometimes depressive symptoms, are a common experience following the death of a child, and should be viewed as normal. However, these experiences may persist for many years or be of such magnitude they prevent normal functioning. Critically, disenfranchised grief, which was prevalent across all countries, was a considerable issue reflected in this review, and a significant source of distress for parents after stillbirth. There was an overwhelming perception that parents and their families felt lonely and abandoned, even by their close relatives. This provides further evidence for the development of interventional programmes which focus on raising understanding and awareness of stillbirth and to address the significant issue of disenfranchised grief, fuelled by underlying stigmatisation of stillbirth.

Overall this review has described the wide-ranging impact of stillbirth. Findings included a few but important positive effects on relationships and a different outlook and approach to life. Findings only reported by mothers included complex responses to their body image after stillbirth, whereas fathers reported the majority of findings related to grief suppression and substance use. The complexity of the findings demonstrates the need for an improvement programme in bereavement care which includes emotional, psychological and financial support for both parents, and for the wider family, and which continues into the subsequent pregnancy. More research is needed to evaluate existing programmes and to focus on the development of new ones. Multiple types of interventions will probably be required in support programmes to meet the multi-factoral issues highlighted and to ensure they can be tailored to individual needs and support the development of any personal growth. Lastly, any improvements to bereavement care will need to be culturally and religiously sensitive and framed around individual cultural beliefs as well as the resource issues in specific country settings.

## Conclusion

Experiencing the birth of a stillborn child is a devastating life-changing event for parents and the wider family. The consequences of stillbirth may vary with parent gender and country. Grief suppression, employment difficulties and financial debt, and increased substance use are particularly prominent in fathers, whereas altered body image and impact on QoL are more specific to mothers. In LMIC stigmatisation, rejection and abuse are widespread.

For parents and families worldwide, stillbirth can have detrimental psychological, physical and social costs, with ongoing effects on interpersonal relationships and subsequently born children. However, when well supported, some parents who experience the tragedy of stillbirth can develop resilience, new life-skills and capacities.

This systematic review of the worldwide literature highlights the need for future investment and research into both stillbirth prevention and aftercare. Such efforts may minimise its negative impact for parents, families and society, and build on any personal development that could be nurtured, if the care provision is timely, appropriate, acceptable, and available.

## Additional files

Additional file 1:
**Demographic information of included studies and intensity effect sizes (IES).** (DOC 298 kb) Additional file 2:
**Thematic sentences, frequency effect sizes (FES) and quotes from extracted findings in the metasummary with all studies included in systematic review.** (DOC 108 kb)
