# Supplementary material for: From grief, guilt pain and stigma to hope and pride – a systematic review and meta-analysis of mixed-method research of the psychosocial impact of stillbirth
Source: BMC Pregnancy Childbirth. 2016 Jan 19;16:9. doi: 10.1186/s12884-016-0800-8 (PMC4719709; doi:10.1186/s12884-016-0800-8)
Supplement: Additional file 1: — Demographic information of included studies and intensity effect sizes (IES). (DOC 298 kb) [file 12884_2016_800_MOESM1_ESM.doc]

| **Continent** | **Author** | **Date** | **Location** | **Participants** | **Topic** | **Design** | **Analysis** | **Sample size** | **Ethnicity** | **Time since SB** | **Gestation at SB (weeks)** | **tIES** | **25%IES** |
| --- | --- | --- | --- | --- | --- | --- | --- | --- | --- | --- | --- | --- | --- |
| Asia | Sun | 2014 | Taiwan  (UMIC) | Bereaved married mothers & fathers | Experiences after stillbirth  Seeing or not seeing the infant | Descriptive  phenomenological approach  In depth interviews | Giorgis method | 24 | Asian | NS | 22, 28, 30, 26, 25, 23, 33, 31, 28, 35, 30, 24 | 5/1110 -  0.45% | 5/780  -  0.64% |
| Asia | Tseng | 2014 | Taiwan (UMIC) | Bereaved married mothers | Recovery after stillbirth | In depth individual interview with purpose full samplings | Inductive analytical, phenomenological | 21 | Asian | NS | 20-37 | 7/1110-  0.63% | 7/780  - 0.9% |
| North America | Gold  (Abstract) | 2014 | USA  (HIC) | Bereaved mothers | Mental health outcomes after stillbirth | Longitudinal survey | Quantitative | 378 bereaved mothers  232 live birth mother | 19% African-American in bereaved group | NS | NS | 1/1110 –  0.09% | 1/780  - 0.13% |
| North America | Huberty | 2014 | USA  (HIC) | Bereaved mothers | Physical exercise after stillbirth | Semi-structured interviews | Thematic analysis | 24 | Caucasian- 91.6%% | 6.33 ±  3.06 month | 20-38 | 7/1110 – 0.63% | 1/780  - 0.13% |
| Africa | Sisay | 2014 | Ethiopia  (LIC) | Grandmothers, mothers – married & unmarried | Attitudes & values around stillbirth & neonatal death | Focus groups | Framework analysis guided by phenomenological approach | 63 grandmothers  74 women  70 unmarried girls | African | NS | 28 + | 13/1110  - 1.2% | 6/780  - 0.77% |
| Europe | Ryninks | 2014 | UK  (HIC) | Bereaved mothers | Experience of contact with stillborn infant | In depth interviews | Interpretive phenomenological analysis-IPA | 21 | White 85.1% | 3/12 after stillbirth | 24+ | 6/1110  - 0.54% | 4/780  - 0.51% |
| Middle East | Mehran | 2013 | Iran  (UMIC) | Mothers | Perinatal loss and maternal-fetal attachment behaviours | Questionnaire, with convenience sampling  MFA scale | Quantitative | 100 pregnant women with or without history of perinatal loss | Arab | NS | NS | 3/1110 –  0.27% | 2/780  - 0.26% |
| Europe | Avelin | 2013 | Sweden  (HIC) | Bereaved mothers & fathers | Grief and relationships after perinatal loss | Postal questionnaire –closed & open questions | Quantitative & content analysis | 55 -  33 mothers  22 fathers | NS | NS | 22- 40 | 6/1110 –  0.54% | 6/780  - 0.77% |
| Europe | Cacciactore | 2013 | Sweden  (HIC) | Bereaved fathers | Fathers’ experience after fetal loss | Questionnaire – open and closed | Quantitative & content analysis | 131 | NS | Majority (n=99) > 2 yrs | 22+ | 4/1110 –  0.36% | 3/780  -  0.38% |
| Europe | Eriandsson | 2013 | Sweden  (HIC) | Bereaved mothers | Seeing & holding a stillborn baby | Online questionnaire | Quantitative | 840 | NS | 0-55 yrs | 22-42 | 1/1110 – 0.09% | 0 |
| Europe | Lindgren | 2013 | Sweden  (HIC) | Bereaved mothers | Experiences after stillbirth | In depth interviews | Content analysis | 23 | NS | 5 weeks – 6 yrs | NS | 2/1110 – 0.18% | 2/780 – 0.26% |
| North America | Welborn | 2012 | USA  (HIC) | Bereaved mothers | Expressing & donating milk after perinatal loss | Semi-structured interviews | Colaizzi’s phenomenological methodology | 21 | NS | NS | NS | 1/1110 –  0.09% | 1/780  - 0.13% |
| North America | Alves | 2012 | USA  (HIC) | Bereaved mother | Meaning following perinatal loss | Constructivist grief therapy | Innovative moments coding system | 1 | African - American | 6 mths | 28 | 2/1110 –  0.18% | 2/780  - 0.26% |
| Australasia | Barr | 2012 | Australia  (HIC) | Bereaved mothers & fathers | Emotion & grief after perinatal loss | Self-reported questionnaire | Quantitative | 126  63 couples | English-Australian 76% | 1 mth | 20+ | 3/1110  - 0.27 | 3/780  - 0.38% |
| Europe | Murphy | 2012 | UK  (HIC) | Bereaved mothers & fathers | Stillbirth, stigma and moral identity | In-depth interviews | Grounded theory analysis | 32  10 couples  12 mothers | Caucasian | 6mths-19yrs | 24+ | 12/1110 –  1.1% | 7/780  -  0.9% |
| Europe | Heazell | 2012 | UK  (HIC) | Bereaved mothers & fathers | Consent process for perinatal postmortem after stillbirth | Online survey – open and closed questions | Quantitative | 460 | Caucasian 95% | 2000 + | 24+ | 1/1110 – 0.09% | 0 |
| Europe | Gravensteen | 2012 | Norway  (HIC) | Bereaved mothers | Long term of stillbirth on depression & QoL | Case/control  Questionnaire  QLI, CES-D,  GHQ-20 | Quantitative | 106  262 live birth mother controls | NS | NS | 23+ or birthweight > 500 grams | 2/1110 –  0.18% | 1/780  - 0.13% |
| Asia | Sutan | 2012 | Malaysia  (UMIC) | Bereaved mothers | Psychosocial impact of stillbirth on muslim women | Focus groups and unstructured interviews | Open coding, thematic analysis | 16 | Asian | NS | 22+ or birthweight > 500 grams | 12/1110 –  1.1% | 10/780  -  1.3% |
| North America | Kelley | 2012 | USA  (HIC) | Bereaved mothers & fathers | Parents & physicians experiences of stillbirthc | Focus groups | Thematic discourse analysis | 18 mothers  2 fathers | NS | NS | NS | 9/1110 –  0.81% | 9/780  -  1.2% |
| Europe | Kersting | 2011 | Germany  (HIC) | Bereaved mothers | Experiences after fetal loss and cognitive behavioural therapy | RCT  Questionnaire | Quantitative | 83 | NS | NS | 2-40 | 1/1110 –  0.09% | 1/780  - 0.13% |
| Europe | Aho | 2011 | Finland  (HIC) | Bereaved fathers | Experiences after fetal loss and intervention | RCT  Questionnaire | Quantitative | 103  bereaved fathers - 62  control- 41 | NS | 6 mths | 20 (or birth weight> 500 grams) -41 | 1/1110 –  0.09% | 1/780  - 0.13% |
| Europe | Eriandsson | 2011 | Sweden  (HIC) | Bereaved mothers | Mothers’ experiences | Online questionnaire | Qualitative content analysis | 515 | NS | 0-41 yrs Av – 5 yrs | 22+ | 14/1110 – 1.3% | 8/780 – 1.0 |
| Europe | Avelin | 2011 | Sweden  (HIC) | Bereaved mothers & fathers | Experiences after stillbirth and sibling support | Focus groups | Content analysis | 27 | NS | 1-22 yrs Av – 6 yrs | 22+ | 12/1110 – 1.1% | 3/780  - 0.38% |
| Europe | Malm | 2011 | Sweden  (HIC) | Bereaved mothers | Experiences after stillbirth | In-depth interviews | Content analysis using inductive method | 21 | NS | 1mth-80mths | 30-42 | 9/1110 - 0.81% | 7/780  - 0.9% - |
| Asia | Gausia | 2011 | Bangladesh  (LIC) | Mothers | Experiences after perinatal loss in LIC | Questionnaire interviews | Quantitative | 476  122 -bereaved m others | Asian | NS | NS | 5/1110 – 0.45% | 5/780 – 0.64% |
| North America | Lang | 2011 | Canada  (HIC) | Bereaved mothers & father | Disenfranchised grief | Interviews | Content analysis | 52- 26 couples | English-Canadian 13% or Other- French, Greek, Italian, Lebanese, Chinese | 2mths, 6 mths & 13 mths | 9 losses < 20 | 6/1110 – 0.54% | 6/780  - 0.77% |
| Europe | Eriandsson | 2011 | Sweden  (HIC) | Bereaved mothers & fathers | Support, stillbirth & grief | Questionnaire | Quantitative | 55  33 mothers  22 fathers | NS | 3mths, 1yr and 2 yr after stillbirth | 22+ | 3/1110 – 0.27% | 2/780  - 0.26% |
| Asia | Yamazaki | 2010 | Japan  (HIC) | Bereaved mothers | Experiences after stillbirth | Interviews | Grounded theory, inductive analysis | 17 | Asian | > 1yr | 28+ | 5/1110 – 0.45% | 2/780  - 0.26% |
| Europe | Vidal | 2010 | Portugal  (HIC) | NA | Pregnancy after perinatal loss | NA | NA | NA | NA | NA | NA | 3/1110 – 0.27% | 1/780  - 0.13% |
| Europe | Radestad | 2010 | Sweden  (HIC) | Bereaved mothers | Advice for pregnancy after stillbirth | Questionnaire | Quantitative | 31 | Nordic | 1 yr | 22+ | 1/1110 – 0.09% | 0 |
| Europe | Eriandson | 2010 | Sweden  (HIC) | 16 mothers and 9 fathers to siblings of stillborn child (surviving twins excluded) 19 siblings, age range 2-12, mean = 7 | Siblings farewell to a stillborn baby | Questionnaire | Quantitative | 44 | Noridic | 3mths & 1 yr | 22+ | 5/1110  - 0.45% | 0 |
| Asia | Sutan | 2010 | Malaysia  (UMIC) | Bereaved mothers | Psychosocial impact of stillbirth | Self-administered questionnaire | Quantitative | 62 | Asian - | 6 wks- 1 yr | NS | 4/1110  - 0.36% | 4/780  - 0.51% |
| Asia | Fottrell | 2010 | Benin  (LIC) | Mothers  after obstetric complications | Psychological distress after obstetric complications | Interview  Questionnaire | Quantitative | 694  near miss & perinatal death = 64 | Asian | 2wks, 6 & 12 mths | NS | 10/1110 –  0.9% | 7/780 – 0.89% |
| North America | Forhan | 2010 | Canada  (HIC) | Bereaved mother | Familys’ journey after perinatal loss | Autoethanography | NS | 1 | NS | Immediately after loss | 37 | 2/1110  -0.18% | 2/780  0  -  0.25% |
| Europe | Turton | 2009 | UK  (HIC) | Mothers | Children subsequent to stillbirth | Interviews  Questionnaires  Observational test | Quantitative | 103  bereaved mothers with subsequent children aged 6-8 yrs - 62 | Caucasian 65.7% | NS | NS | 3/1110  - 0.27% | 0 |
| North America | Armstrong | 2009 | USA  (HIC) | Bereaved mothers & fathers | Psychological distress after the birth of a health child after perinatal loss | Interview  Questionnaires  - CES-D, IES | Quantitative | 72  - 36 couples | Caucasian 95.5% | 3^rd^ trimester, 3-6mths & 6-8mths pospartum | NS | 9/1110  - 0.81% | 3/780  -  0.38% |
| Europe | Radestad | 2009 | Sweden  (HIC) | Bereaved mothers | Long-term outcomes & holding their stillborn baby | Questionnaire. STAI-S, STAI-T, CES-D | Quantitative | 309 | NS | NS | 28-42 | 2/1110  -0.18% | 1/780  -  0.13% |
| North America | Cacciatore | 2009 | USA  (HIC) | Bereaved mothers | Social support and maternal anxiety & depression | Questionnaire -HSCL | Quantitative | 769 | Caucasian – 88.6% | 18 mths | NS | 4/1110  -  0.36% | 4/780  -  0.51% |
| Europe | Turton | 2009 | UK  (HIC) | Mothers | Long term sequelae of stillbirth | Case-control  Structured clinical interview for DSM IV | Quantitative | 103  - 52 bereaved mothers | Caucasian -65.4% | 7 yrs after pregnancy subsequent to stillbirth | 18 + | 5/1110  -  0.45% | 2/780  -  0.25% |
| Europe | Surkan | 2009 | Sweden  (HIC) | Bereaved mothers | Social support | Postal questionnaire and CES-D | Quantitative | 314 | Nordic | 3 yrs | 28+ | 6/1110  -  0.54% | 6/780  -  0.77% |
| Africa | Adeyemi | 2008 | Nigeria  (LIC) | Mothers | Depression after perinatal loss | Interview  Questionnaire – HAD, EDPS | Quantitative | 108 -54 bereaved mothers, 54 controls | NS | Immediately after loss | NS | 4/1110  -  0.36% | 3/780  -  0.38% |
| North America, Europe | Cacciatore | 2008 | Canada, USA, UK (HIC) | Bereaved mothers | Contact with stillborn babies & maternal depression & anxiety | Questionnaire -HSCL | Quantitative | 2,900 | Caucasian – 90.1% | < 1yr – 3+ yrs | NS | 2/1110  -  0.18% | 1/780  -  0.13% |
| Europe | Surkan | 2008 | Sweden  (HIC) | Bereaved mothers | Events after stillbirth in relation to depression | Questionnaire  CES-d | Quantitative | 380 | NS | In 1991 | 28+ | 2/1110  -  0.18% | 2/780  -  0.26% |
| North America | Barr | 2008 | USA  (HIC) | Bereaved mothers | Fear of grief & death in bereaved mothers | Online questionnaire – PGS -33, MFODS | Quantitative | 400 | European  American (n = 288, 72%), African American (n =6, 1.5  %),Native  American (10,= 2.5  %), Asian American (n=3, 0.8  %),Hispanic(n =  13, 3.3  %), and other(non-American) (n=80, 20  %) | 0-343 mths av = 8 | NS | 1/1110  -  0.09% | 0 |
| Europe | Pidgeon | 2007 | UK  (HIC) | Bereaved father | Life after stillbirth | Narrative- N/A | N/A | 1 | NS | NS | NS | 3/1110  -  0.27% | 1/780  -  0.13% |
| North America | Cacciatore | 2007 | USA  (HIC) | Bereaved mothers | Mothers’ experience after stillbirth | Qualitative and open narrative postal questionnaire | Qualitative, phenomenological approach | 47 | 38 European Americans - 3 African Americans - 3 Latino -2 Other -1 | 1- 10yrs | 20-37 | 3/1110  -  0.27% | 1/780  -  0.13 |
| Europe | Turton | 2006 | UK  (HIC) | Mothers & fathers | Impact of stillbirth on fathers in subsequent pregnancy | Interviews  Questionnaire – BDI, EPDS, SSDI, PTSD-I, GRIMS | Quantitative | 200-  37 - controls  32 bereaved couples in subsequent pregnancy | Caucasian 72.4%  African 11.8%  Asian 10.5% | 11-44 mths av – 18.8 | 20-41 | 4/1110  -  0.36% | 4/780  -  0.51% |
| North America | O’Leary | 2006 | USA  (HIC) | Bereaved fathers | Fathers’ perspectives after stillbirth | Interviews  Descriptive phenomenology | Giorgis method of analysis | 10 | NS | Within 1 yr | NS | 17/1110  - 1.5% | 11/780  -  1.4% |
| Australasia | Barr | 2006 | Australia  (HIC) | Bereaved mothers & fathers | Grief & subsequent pregnancy | Semi-structured interviews and PGS questionnaire | Quantitative | 126  63 couples-31 bereaved by stillbirth | English-Australian – 76% | 1mth & 13mth FU | 20+ | 2/1110  -  0.18% | 1/780  -  0.13 |
| Europe | Saflund | 2006 | Sweden  (HIC) | Bereaved mothers & fathers | Experiences after stillbirth | Questionnaire and Well-Being Questionnaire | Quantitative | 55  33 mothers  22 fathers | Nordic | 3 mths | 22-43 | 6/1110  - 0.54% | 4/780  -  0.51% |
| North America | Armstrong | 2004 | USA  (HIC) | Bereaved mother & fathers in subsequent pregnancy | Perinatal loss & subsequent pregnancy | Cross-sectional survey – IES, CES-D, PAI | Quantitative | 80 – 40 couples | Caucasian 93% | NS | Av – 22.6 | 7/1110  - 0.63% | 7/780  -  0.9% |
| North America | Cote-Arsenault | 2004 | USA  (HIC) | Bereaved mothers & fathers | Support groups & pregnancy loss | Observation of support groups, individual interviews, postal surveys | Quantitative & qualitative | 26 –support group  23- women  3- men  12- interviews  130 – postal surveys | 90% white, 10% minorities (mainly Pacific Islanders) | NS | NS | 15/1110  -  1.35 % | 15/780  -  1.92% |
| Europe | Saflund | 2004 | Sweden  (HIC) | Bereaved mothers & fathers | Role of care givers after stillbirth | Interviews | Qualitative content analysis | 57 | NS | 4-6 yrs | 28+ | 3/1110  -  0.27% | 3/780  -  0.38% |
| Europe | Avelin | 2014 | Sweden  (HIC) | 13 - 17 year old Bereaved adolescent half-siblings to stillborn child | Half-siblings & stillbirth | Interviews | Content analysis | 13 | NS | NS | NS | 19/1110  -  1.71% | 5/780  -  0.64% |
| North America | Fenstermacher | 2014 | USA  (HIC) | Bereaved mothers | Experience of perinatal loss in Black adolescents | Interview- face to face or phone | Qualitative – constant comparative | 19 | African-American | NS | 9-32 Av- 19.8 | 5/1110  -  0.45% | 4/780  -  0.51% |
| Europe | Radestad | 2014 | Sweden  (HIC) | Bereaved mothers | Mothers’  experiences | In-depth interviews | Qualitative content analysis with inductive approach | 26 | NS | <1yr- 6 yrs | 28+ | 1/1110  -  0.09% | 1/780  -  0.13% |
| North America | O’Leary | 2013 | USA  (HIC) | Bereaved mothers & fathers & subsequent siblings | Contact with the baby after stillbirth | Interviews | Secondary thematic analysis | 9 - elderly bereaved mothers & fathers  7- subsequent siblings | NS | 50-70 yrs | NS | 4/1110  -  0.36% | 3/780  -  0.38% |
| Europe | Downe | 2013 | UK  (HIC) | Bereaved mothers & fathers | Experience in UK hospitals | Qualitative in-depth interviews (face-to-face or telephone) | Constant comparative technique from grounded theory | 25 | NS | 1-9 yrs | 24-42 | 22/1110  -  1.98% | 14/780  -  1.79% |
| Europe | Murphy | 2012 | UK  (HIC) | Bereaved mothers & fathers | Finding the positive in perinatal loss | In-depth interviews | Grounded theory | 32-  10 couples  12 mothers | Caucasian 30/32 | 6mths- 17 yrs | 24+ | 15/1110  -1.35% | 3/780  -  0.38% |
| Europe | Anderson | 2012 | Sweden  (HIC) | Bereaved mothers & fathers and surviving twins | Bereavement and twins | Interviews | Thematic analysis | 8-  4 parents & 4 surviving twins | NS | 35 yr | NS | 17/1110  -  1.53% | 4/780  -  0.51% |
| Europe | Nordlund | 2012 | Sweden  (HIC) | Bereaved mothers | Psychosocial care after perinatal loss | Online questionnaire | Content analysis | 213 | NS | 0-2yrs | 22+ | 19/1110  -  1.71% | 15/780  -  1.92% |
| Europe | Kerslake | 2012 | UK  (HIC) | Bereaved mothers | Learning from loss | Narrative-NA | NA | 2 | Caucasian | NS | 40+ | 11/1110  -  0.99% | 7/780  -  0.90% |
| Australasia | Lee | 2012 | Australia  (HIC) | Bereaved mothers | Experiences after pregnancy loss | Online open ended questionnaire | Thematic analysis | 14 | NS | 3-4mths | 20-37 | 7/1110  -  0.63% | 2/780  -  0.26% |
| Australasia | Warland | 2011 | Australia (HIC) | Bereaved mothers & fathers | Parenting after infant loss | Informal interviews | Thematic analysis | 13 | NS | 4-18yrs | NS | 17/1110  - | 6/780  -  0.77% |
| Australasia, North America | O’Leary | 2011 | Australia, USA (HIC) | Bereaved mother, fathers & grandparents | Bereaved parents & grandparents after fetal loss | Interviews | Thematic analysis | 32 | Caucasian | 1-10yrs | NS | 23/1110  -  2.07% | 14/780  -  1.79% |
| Europe | Dyregov | 2011 | Norway  (HIC) | NS | Sexuality after stillbirth | Questionnaire and interview (10 couples interviewed) | Quantitative & qualitative | 285 | NS | NS | NS | 55/1110  -  4.95% | 25/780  -  3.21% |
| Asia | Sun | 2011 | Taiwan  (UMIC) | Bereaved mothers | Motherhood after pregnancy loss | Interviews | Interpretive phenomenological analysis- IPA | 6 | Asian | 2-3.5 yrs | 10-37 | 10/1110  -  0.9% | 7/780  -  0.90% |
| Europe | Wood | 2011 | UK  (HIC) | Bereaved mothers | Stressful life events and pregnancy loss | Interviews  Questionnaire – HADS, LTE-Q | Quantitative & qualitative- framework analysis | 200 | NS | NS | NS | 3/1110  -  0.27% | 3/780  -  0.38% |
| North America | Cote-Arsenault | 2011 | USA  (HIC) | Bereaved mothers in subsequent pregnancy | Pregnancy after perinatal loss | Mixed methods  Self-reported questionnaire- PAS | Quantitative  Content & thematic | 63 | Caucasian -84% | NS | NS | 6/1110  -  0.54% | 6/780  -  0.77% |
| North America | Capitulo | 2010 | USA  (HIC) | Bereaved mothers & fathers | Measuring perinatal grief | Questionnaire | Quantitative | 90-  50 bereaved parents, 40 controls | NS | 1 yr | NS | 2/1110  -  0.18% | 2/780  -  0.26% |
| Europe | Gaudet | 2010 | France  (HIC) | Mothers in subsequent pregnancy | Pregnancy after perinatal loss | Online of face to face - questionnaires – HADS, PGS, PSEQ | Quantitative | 170-  96 – bereaved mothers  74- controls | NS | NS | NS | 3/1110  -  0.27% | 3/780  -  0.38% |
| North America | Cacciatore | 2010 | USA  (HIC) | Bereaved mothers | Experiences of women & family after stillbirth | Self-administered postal questionnaire - qualitative and open-ended narrative | Thematic analysis | 47 | NS | <1 year n= 10 1-2 years n= 10 2-5 years n=17 5-10years n=7 >10 years n = 3 | 40 | 14/1110  -  1.26% | 13/780  -  1.67% |
| Asia | Hiruta | 2009 | Japan  (HIC) | Bereaved mothers | Grieving processes | Interviews | Thematic analysis | 5 | Asian | NA | NA | 19/1110  -  1.71% | 12/780  -  1.54% |
| Europe | Radestad | 2009 | Sweden  (HIC) | Bereaved mothers | Holding a stillborn baby | Questionnaires | Quantitative | 33 | NS | 3mths | 22+ | 2/1110  -  0.18% | 1/780  -  0.13% |
| North America | Cacciatore | 2008 | USA  (HIC) | 16 married or cohabiting heterosexual couples + 56 conference attendees | Stillbirth & the couple | Questionnaire, Discussion Group, Narrative responses submitted in writing | Thematic analysis, Constant comparison method | 87 | NS | NS | NS | 29/1110  -  2.61% | 23/780  -  2.95% |
| North America | Cote-Arsenault | 2007 | USA  (HIC) | Bereaved mothers in subsequent pregnancy | Pregnancy following perinatal loss | Qualitative study of  pregnancy calendar entries and field notes. | Thematic analysis | 69 | Caucasian 88.4% | NS | NS | 5/1110  -  0.45% | 5/780  -  0.64% |
| North America | Kavanaugh | 2004 | USA  (HIC) | Bereaved mothers & fathers | Social support & perinatal loss | Secondary analysis. Open-ended interview (5 mothers, 3 fathers) Open-ended interviews (17 mothers, 6 fathers) | Colaizzi's approach,Vaux's theory. Concept analysis/thematic analysis | 31 -  22 Mothers 9 Fathers | NS | 4-15 | 16-23 | 12/1110  -  1.08% | 11/780  -  1.41% |
| Europe | McCreight | 2004 | UK  (HIC) | Bereaved fathers | Males perspective of pregnancy loss | Observations of self-help groups & semi-structured interviews | ?Thematic analysis | 14 | NS | 2mths-20yrs | 8-40 | 12/1110  -  1.08% | 5/780  -  0.64% |
| Asia | Hsu | 2004 | Taiwan  (UMIC) | Bereaved mothers | Interpretations of stillbirth | Interviews – interpretive ethanographic | Thematic analysis | 20 | Asian | Within 1 yr | 20+ | 7/1110  -  0.63% | 6/780  -0.77% |
| North America | Pector | 2004 | USA, Canada  (HIC) | Bereaved mothers & fathers | Bereavement in multiple-birth | Online survey | Quantitative & qualitative – grounded theory approach | 70 | NS | 1980-2001 | NS | 10/1110  -  0.90% | 4/780  -  0.51% |
| North America | Lang | 2004 | Canada  (HIC) | Bereaved mothers & fathers | Health after perinatal loss | Longitudinal. Lang and Goulet Hardiness Scale. Support Behaviors Inventory, Family Adaptability and Cohesion Evaluation Scale, ENRICH Marital Satisfaction scale, Subjective Appraisal Ratings of Stressors, PGS | Quantitative | 214 | NS | 2mths | NS | 7/1110  -  0.63% | 6/780  -  0.77% |
| North America | Kavanaugh | 2005 | USA  (HIC) | Bereaved mothers & fathers | Perinatal loss in low income African Americans | Interviews | Qualitative – adapted from Colaizzi | 23-  17 mothers & 6 partners | African- Americans | NS | 17-37 | 13/1110  -  1.17% | 7/780  -  0.90% |
| Australasia | St John | 2006 | Australia  (HIC) | Bereaved mothers | Experience of perinatal loss | Unstructured interviews | Content analysis | 3 | NS | NS | NS | 13/1110  -  1.17% | 11/780  -  1.41% |
| North America | Schiff | 2006 | USA  (HIC) | Women who were hospitalised postpartum for a suicide attempt | Risk of suicide and perinatal loss | Case- control note review | Quantitative | 520 women & 2204 controls | Caucasian- 75% | NS | NS | 2/1110  -  0.18% | 2/780  -  0.26% |
| North America | Cote-Arsenault | 2006 | USA  (HIC) | Bereaved mothers in next pregnancy | Experiences after fetal loss | Pregnancy calendars, Qualitative descriptive design, complete quant. instruments, field notes from researchers | Thematic analysis and content analysis | 82 | Caucasian 86.7% | 3.5-40 weeks | NS | 21/1110  -  1.89% | 17/780  -  2.18% |
| North America | Cote-Arsenault | 2007 | USA  (HIC) | Bereaved mothers | Pregnancy after perinatal loss | Questionnaire – SiL, MTI, MAACL-R, PAS, WCCL-R | Quantitative | 82 | Caucasian = 88% | Av – 2.3 yrs | Av – 11.1 | 2/1110  -  0.18% | 2/780  -  0.26% |
| North America | Cote-Arsenault | 2003 | USA  (HIC) | Pregnant women 17-28 weeks | Anxiety & perinatal loss | Questionnaire- PAS, LOT, SSA scale | Quantitative | 160 women  who were - 96  multigravidwith no history of loss and 74 women  with a history of one or two losses. | Caucasian – 91% | 1-12yrs  Av 2.93 | Av – 10.38 | 3/1110  -  0.27% | 2/780  -  0.26% |
| North America | Hazen | 2003 | USA  (HIC) | Bereaved mothers | Disenfranchised grief | Interviews | Thematic analysis | 14 | Caucasian- 12  African-American - 2 | 1965-199 | NS | 2/1110  -  0.18% | 1/780-  -  0.13% |
| Asia | Hsu | 2002 | Taiwan  (UMIC) | Bereaved mothers | Adaption to stillbirth | Interviews- interpretive ethano graphic approach | Qualitative according to Agar | 20 | Asian | Within 2 yrs | NS | 6/1110  -  0.54% | 6/780  -  0.77% |
| Europe | Kitson | 2002 | Sweden  (HIC) | Bereaved fathers | Fathers’ experience after stillbirth | Interviews | Thematic analysis | 11 | NA | NA | 32-42 | 2/1110  -  0.18% | 1/780-  -  0.13% |
| Europe | Radestad | 2001 | Sweden  (HIC) | Mothers | Long term effects of stillbirth | Questionnaire | Quantitative | 759  314- bereaved mothers, 322 -controls | NA | NA | NA | 8/1110  -  0.72% | 8/780  -  1.03% |
| North America | Sanchez | 2001 | Sweden  (HIC) | Bereaved mothers | Support after perinatal loss | In-depth semi-structured interviews | Qualitative | 12 | NS | 1992-1995 | 20+ | 2/1110  -  0.18% | 1/780-  -  0.13% |
| North America | Armstrong | 2001 | USA  (HIC) | Bereaved fathers with partner currently pregnant | Fathers’ experiences of pregnancy after loss | Phenomenological. Unstructured in-depth interviews | Thematic analysis | 4 | 3Caucasian, 1 Black Jamaica | < 2yrs | 12-20 | 8/1110  -  0.72% | 6/780  -  0.77% |
| Europe | Sansoni | 2001 | Italy  (HIC) | Bereaved mothers & fathers | Grief after perinatal loss | Questionnaire – HGRC, BDI-II | Quantitative | NA | NA | NA | NA | 1/1110  -  0.09% | 0 |
| North America | Cote-Arsenault | 2000 | USA  (HIC) | Bereaved mothers in subsequent pregnancy | Pregnancy after perinatal loss | Qualitative inquiry, focus group or interview (both using interview guide) | Thematic analysis | 13 | Caucasian = 11, Asian/Pacific Islander = 1, Hispanic = 1 | NS | 8-37 | 22/110  -  1.98% | 21/780  -  2.70% |
| North America | Grout | 2000 | USA  (HIC) | 7 Bereaved families: 3 couples, 1 father, 3 mothers | Replacement child after perinatal loss | Interviews –open ended | Grounded theory approach, constant comparative method | 10 | Caucasian | 2-10yrs | NS | 1/1110  -  0.09% | 1/780  -  0.13% |
| North America | Schreiber | 2000 | Canada  (HIC) | Bereaved mothers & fathers | Experiences after perinatal loss | Interviews- semi-structured | Qualitative | 22 -  16 women  6 men | NS | NS | 20+ | 1/1110  -  0.09% | 1/780  -  0.13% |
| Australasia | Vance | 2002 | Australia  (HIC) | Bereaved mothers & fathers | Couple distress after perinatal loss | Interviews | NS | 138 | NS | 2, 8, 15 & 30 mths | NS | 12/1110  -  1.08% | 12/780  -  1.54% |
| Europe | Doug | 2010 | UK  (HIC) | Bereaved fathers | PTSD & perinatal loss | Self-completion questionnaires, validated tools to screen and diagnose PTSD, depression and anxiety. | Quantitative | 150 | NS | 4 days & 4 wks post delivery | NS | 1/1110  -  0.09% | 1/780  -  0.13% |
| Australasia | Swanson | 2002 | Australia  (HIC) | Mothers where 1 twin or higher multiple died | Multiple pregnancies and perinatal loss | Interviews, BDI PGS(Short Version), Focus Groups | Quantitative & thematic analysis | 66 | Caucasian | 0-1 year = 3, 2-5y = 8,   6-10y = 22,   11-15y =  19,   16-20y = 12,  21-41y = 2 | NS | 18/1110  -  1.62% | 14/780  -  1.79% |
| Africa | Van der Sijpt | 2014 | Cameroon  (LMIC) | NS | Pregnancy loss in Cameroon | Observation and discursive strategies | NS | NS | Cameroon - Gbigbil | NS | NS | 3/1110  -  0.27% | 1/780  -  0.13% |
| Europe | Christiansen | 2013 | Denmark  (HIC) | Bereaved mothers & fathers | PTSD & perinatal loss | Questionnaire package. Harvard Trauma Questionnaire, CSQ, Crisis Support Scale, Revised Adult Attachment Scale | Quantitative | 634 | NS | 1.2mths-18yrs Av – 3.4 yrs | 22+ | 2/1110  -  0.18% | 2/780  -  0.26% |
| Europe | Breines | 2013 | Norway  (HIC) | Bereaved mothers | Grief & anxiety after perinatal loss | Questionnaire | Quantitative | 2753 | NA | NA | NA | 2/1110  - 0.18% | 2/780  -  0.26% |
| North America & NS | Gold | 2012 | USA(HIC) & NS | Bereaved mothers | Internet message boards for pregnancy loss | Online survey including one open-ended question | Quantitative & summary descriptive statistics | 1006 | Caucasian | NS | NS | 8/1110  -  0.72% | 4/780  -  0.51% |
| Europe | Blackmore | 2011 | UK  (HIC) | Bereaved mothers in subsequent pregnancy | Previous loss & depression | Questionnaire | Quantitative | 13,133 | NS | NS | NS | 4/1110  -  0.36% | 4/780  -  0.51% |
| North America | Hutti | 2011 | USA  (HIC) | Bereaved mothers | Pregnancy following perintal loss | Longitudinal cohort study. Telephone interview. CES-D, Spielberger State-Trait Anxiety Scale, Pregnancy Outcome Questionnaire, IES, Maternal Attitude Questionnaire, also healthcare utilisation | Quantitative | 32 | African-American - 2.8% White -93.1% Hispanic/Latino - 2.8% Asian - 1.4% | NS | NS | 1/1110  -  0.09% | 0 |
| North America | Cowchock | 2010 | USA  (HIC) | Bereaved mothers | Religious beliefs and perinatal loss | Religious questionnaire, PGS | Quantitative | 110 | NS | 4-6 weeks & 1 yr | NS | 4/1110  -  0.36% | 3/780  -  0.38% |
| North America | Gold | 2010 | USA  (HIC) | Bereaved mothers & fathers | Relationships after perinatal loss | Data from National Survey of Family Growth | Quantitative | 7770 | NS | NS | 20+ | 1/1110  -  0.09% | 1/780  -  0.13% |
| Europe | Luczak | 2010 | Poland  (HIC) | Bereaved mothers | Early & late effects of pregnancy loss | NA | NA | NA | NA | NA | NA | 1/1110  -  0.09% | 1/780  =  0.13% |
| Australasia, North America, Europe | Peel | 2010 | Australia, Canada, UK, USA  (HIC) | Non-heterosexual bereaved mothers | Pregnancy loss in lesbian & bisexual women | Online survey | Quantitative & thematic analysis | 60 | NS | NS | 24+ | 8/1110  -  0.72% | 8/780  -  1.03% |
| South America | Couto | 2009 | Brazil  (UMIC) | Bereaved mothers in next pregnancy | Psychological symptoms in pregnancy after perinatal loss | Interviews, Short Form 36 QoL questionnaire, Depression & Anxiety Scale | Quantitative | 120 | Caucasian - 73 | NS | NS | 2/1110  -  0.18% | 2/780  -  0.26% |
| Africa | Obi | 2009 | Nigeria  (LMIC) | Bereaved mothers | Depression following pregnancy loss in Nigeria | Questionnaire survey. Zung self-rating Depression Scale | Quantitative | 202 | African | < 3mths | NS | 2/1110  -  0.18% | 2/780  -  0.26% |
| North America | Price | 2008 | USA  (HIC) | Mothers | Parenting capacity after loss | ECLS-B data, incl. modified CES-D, Nursing Child Assessment Teaching Scale | Quantitative | 10,688 | NS | NS | NS | 2/1110  -  0.18% | 1/780  -  0.13% |
| Europe | McCreight | 2008 | UK  (HIC) | Bereaved mothers | Perinatal loss | In-depth interviews | Content & thematic analysis | 23 | NS | <3 years = 18, >3 years = 2, >5 years = 3 | NS | 11/1110  -  0.99% | 10/780  -  1.28% |
| North  America | Armstrong | 2007 | USA  (HIC) | Bereaved mothers & fathers | Psychological symptoms in pregnancy after perinatal loss | Telephone interviews, Impact of the Event Scale, CES-D Scale | Quantitative | 36 | NS | NS | Av – 22.2 | 11/1110  -  0.99% | 11/780  -  1.41% |
| North America | Barr | 2007 | USA  (HIC) | Bereaved mothers | Emotions & grief | Questionnaire (online or download). Dispositional Envy Scale, Interpersonal Jealousy Scale, Personal Feelings Questionnaire-2, PGS-33 | Quantitative | 441 | European-American - 70.5% African-American- 1.8% Native American- 2.5% Asian-American-0.7% Hispanic-3.4% Other (Non-Am.)-21.1% | 0-343 mths  Median – 8mths | NS | 1/1110  -  0.09% | 1/780  -  0.13% |
| Europe | Pantke | 2006 | UK  (HIC) | University students whose parents experienced perinatal loss when participant was 5yrs or younger. | Young adults whose parents had experienced pregnancy loss | Parent Bonding Instrument. Rosenberg Self-Esteem Scale. Mental Health Index 5 | Quantitative | 77 | NS | 13-18 yrs | NS | 5/1110  -  0.45% | 0 |
| North America | Coleman | 2005 | USA  (HIC) | Bereaved mothers | Child maltreatment & perinatal loss | Interviews/ Observation | Quantitative | 133 | NS | NS | NS | 4/1110  -  0.36% | 0 |
| Europe | Jind | 2003 | Denmark  (HIC) | Bereaved mothers & fathers | Parents adjustment to loss | Questionnaire | Quantitative | 110 | NS | 1-4 wks & 1 yr | 15-22 | 13/1110  -  1.17% | 13/780  -  1.67% |
| North America | Van | 2003 | USA  (HIC) | Mothers | Grief after pregnancy loss | Semi-structured interviews. Grounded theory method. | Constant comparative | 20 | African-American | < 3yrs Av -2 | 1^st^ trimester majority | 16/1110  -  1.44% | 1/780  -  1.15% |
| Europe | Lee | 2002 | UK  (HIC) | 1 mother, 1 psychotherapist who provides examples | Grief in future pregnancy after perinatal loss | Narrative | NS | >2 | NS | NS | 37+ | 14/1110  -  1.26% | 7/780  -  0.90% |
| North America | Franche | 2001 | Canada  (HIC) | Bereaved mothers | Grief in future pregnancy after perinatal loss | Questionnaire- PGS, Depressive Experiences Questionnaire - Self-Criticism Subscale, Abbreviated Dyadic Adjustment Scale | Quantitative analysis | 110 | NS | < 4 yrs Av – 15.1 mths | 4-42 Av – 17.5 | 3/1110  - 0.27% | 3/780  -  0.38% |
| North America | Van | 2001 | USA  (HIC) | Bereaved mothers | African-american women after perinatal loss | Individual interviews, open-ended questions | Grounded theory methodology, thematic analysis | 10 | African- american | Av – 4 yrs | NS | 7/1110  - 0.63% | 5/780  -  0.64% |
| Europe | Wilson | 2001 | UK  (HIC) | 8 families (5 couples, 3 mothers) with at least one other child at time of loss | Support fro siblings after perinatal loss | Semi-structured interviews | McLeod's stages, concept analysis | 13 | NS | < 6 yrs | NS | 10/1110  -  0.90% | 3/780  -  0.38% |
| Europe | Bernazzani | 2003 | UK  (HIC) | Bereaved mothers & sisters | Vulnerability factors for depression | Interviews. Present State Examination, Childhood Experience of Care and Abuse, Adult Life Phase Interview, Pregnancy Birth Ratings, Marital Ratings | Quantitative | 198 | NS | NS | NS | 3/1110  -  0.27% | 3/780  -  0.38% |
| North America | Hopkins Hutti | 2014 | USA  (HIC) | Bereaved mothers in subsequent pregnancy | Grief in subsequent pregnancy after perinatal loss | Online Pregnancy Outcome Questionnaire, IES, CES-D, Autonomy and Relatedness Inventory, PGIS | Quantitative | 227 | NS | NS | NS | 3/1110  - 0.27% | 3/780  -  0.38% |
| North America | Varney | 2014 | USA  (HIC) | Bereaved mother & father | Perinatal loss | Narrative / Psychotherapist's notes | NS | 2 | Caucasian | NS | NS | 16/1110  -  1.44% | 9/780  -  1.15% |
| North America | Shreffler | 2011 | USA  (HIC) | Bereaved mothers | Distress & Pregnancy loss | Questionnaire | Quantitative | 1284 | Caucasian 60%  Black 15%  Hispanic 23%  Other 0.02% | 0.05 -25.12 yrs  Av 11.13yrs | NS | 5/1110  -  0.45% | 5/780  -  0.64% |
| NS | Froen | 2011 | NS | Bereaved mothers & fathers | Why stillbirths matter | Online survey | Quantitative | 3617 | NS | NS | 22+ | 6/1110  -  0.54% | 4/780  -  0.51% |
| North America | Jaffe | 2011 | USA  (HIC) | Psychotherapist | Experience of stillbirth | Narrative | NA | 1 | NS | NS | NS | 19/1110  -  1.71% | 17/780  -  2.18% |
| Europe | Reid | 2007 | UK  (HIC) | Bereaved mothers & fathers | Pregnancy loss & subsequent pregnancy | Narrative | NA | 4 | NS | NS | 19-40 | 19/1110  -  1.71% | 14/780  -  1.79% |
| Europe | Hughes | 2006 | UK  (HIC) | Bereaved mothers | Infant disorganisation after perinatal loss | Questionnaire, Adult Attachment Interview, EPDS, Spielberger State-Trait Inventory, PTSD-I Interview, Strange Situation procedure | Quantitative | 31 | NS | NS | 18+ | 2/1110  -  0.27% | 2/780  -  0.26% |
| Europe | Turton | 2004 | UK  (HIC) | Bereaved mothers in subsequent pregnancy | PTSD & stilllbirth | Cohort study. Demographic info questionnaire. PTSD-I Interview, Adult Attachment Interview, Strange Situation assessment of infant security. | Quantitative | 60 | NS | NS | NS | 4/1110  -  0.36% | 1/780  -  0.13% |
| Europe | Hughes | 2001 | UK  (HIC) | Children subsequent to stillbirth | Infants born subsequent to stillbirth | Detailed interview. Demographic questionnaire. AAI, EPDS, SSTI, Beck Depression Inventory, Strange Situation test | Quantitative | 53 (+ 53 control infants) | Caucasian - 34 (64%), Afro-Caribbean - 6 (11%), Indian/Pakistani - 9 (17%), African - 3 (6%), Chinese - 1 (2%) | 11mths- 186mths Av- 18mths | <28 weeks -31 (56%) >27 weeks - 24 (44%) | 3/1110  -  0.27% | 1/780  -  0.13% |
| North America | Hogue | 2015 | USA  (HIC) | Bereaved Mothers | Stillbirth & depression | Telephone interview, use of psychosocial instruments. EDS, Spielberger Trait Anxiety Scale, Stressful Life Events Scale | Quantitative | 275  (+ 522 controls live-birth) | Caucasian - 45.1%  Black – 18.8%  Hispanic – 29.9% | 6-36 mths | 18- > 37 | 3/1110  -  0.27% | 3/780  -  0.38% |
| North America | Gold | 2014 | USA  (HIC) | Bereaved mothers | Anxiety & OCD after perinatal loss | GAD-7, MINI-SPIN, PRIME-MD PHQ Panic Module, Obsessive Compulsive Inventory, PHQ-8, MOS-SSS, plus intimate partner violence | Quantitative | 377  (+ 232 controls live-birth) | Caucasian – 76%African America -19% | Median – 9mths | NS | 5/1110  -  0.45% | 5/780  -  0.64% |
| North America | Tran | 2014 | USA  (HIC) | Bereaved mothers | Smoking & pregnancy loss | Perinatal records extracted from NSW Perinatal Data Collection and NSW Admitted Patient Data Collection | Quantitative | 1144 | NS | NS | NS | 4/1110  -  0.36% | 0 |
| North America | Lacasse | 2014 | USA  (HIC) | Bereaved mothers & fathers | Psychiatric medication after pregnancy loss | Observational study, online survey of prescribed medication | Quantitative & life table analysis | 235 | Caucasian – 82.3% | NS | 20+ | 8/1110  -  0.72% | 8/780  -  1.03% |
| Europe | Newitt | 2014 | UK  (HIC) | Bereaved mothers & fathers | Spiritual support and perinatal loss | Narrative/chaplains notes | NA | 8 | NS | NS | NS | 6/1110  -  0.54% | 5/780  -  0.64% |
| Europe | Brierley-Jones | 2015 | UK  (HIC) | Bereaved mothers | Stillbirth & stigma | Online questionnaire: Making and Sharing Memories Questionnaire. Free-text responses to open-ended questions. DASS-21, Posttraumatic Stress Symptom Scale. | Thematic & Content analysis | 162 | Caucasian – 95% | < 10yrs 0.25 -120mths | 20-43 | 31/1110  -  2.82% | 15/780  -  1.92% |
| Asia | Takaki | 2014 | Japan  (HIC) | Women undergoing fertility treatment (not all with previous perinatal loss) | Psychological symptoms and fertility treatment | Postal questionnaire. Kessler 6 question Psychological Distress Scale. | Quantitative | 635 | NS | NS | NS | 4/1110  - 0.36% | 2/780  -  0.26% |
| Europe | Munk-Olsen | 2014 | Denmark  (HIC) | Cases of perinatal loss | Psychiatric disorders after perinatal loss | Population-based cohort study. Data from nationwide population registers | Quantitative | 87,687 | NS | 12mths | NS | 7/1110  -  0.63% | 7/780  -  0.90% |
| North America | Hutti | 2015 | USA  (HIC) | Bereaved mothers in subsequent pregnancy | Subsequent pregnancy after perinatal loss | Cross-sectional online survey. Pregnancy Outcome Questionnaire, IES, CES-D, ARI, PGIS | Quantitative | 227 | Caucasian – 78%  Other – 22% | NS | 20+ | 5/1110  -  0.45% | 5/780  -  0.64% |
| Europe | Christiansen | 2014 | Denmark  (HIC) | Bereaved mothers & fathers | Bereavement and sex differences after perinatal loss | Cross-sectional study, written questionnaire. HTQ Part IV, Revised Adult Attachment Scale, Coping Styles Questionnaire, Crisis Support Scale | Quantitative | 634 | NS | 1.2 mth – 18yrs  Av – 3.4yrs | 13-44 Av- 34.5 | 6/1110  -  0.54% | 6/780  -  0.77% |

tIES – total Intensity Effect Size – number of findings in paper divided by total number of findings

25%IES - 25% Intenisty Effect Size – number of findings in paper with FES > 25% divided by total number of findings with FES > 25%
