# Supplementary material for: From grief, guilt pain and stigma to hope and pride – a systematic review and meta-analysis of mixed-method research of the psychosocial impact of stillbirth
Source: BMC Pregnancy Childbirth. 2016 Jan 19;16:9. doi: 10.1186/s12884-016-0800-8 (PMC4719709; doi:10.1186/s12884-016-0800-8)
Supplement: Additional file 2: — Thematic sentences, frequency effect sizes (FES) and quotes from extracted findings in the metasummary with all studies included in systematic review. (DOC 108 kb) [file 12884_2016_800_MOESM2_ESM.doc]

**References included in systematic review**

Adeyemi, A., Mosaku, K., Ajenifuja, O., Fatoye, F., Makinde, N., & Ola, B. Depressive symptoms in a sample of women following perinatal loss. Journal Of The National Medical Association, 2008: 100 ; 1463–1468.

Aho, A. L., Tarkka, M.-T., Astedt-Kurki, P., Sorvari, L., & Kaunonen, M. Evaluating a bereavement follow-up intervention for grieving fathers and their experiences of support after the death of a child--a pilot study. Death Studies, 2011: 35; 879–904.

Alves, D., Mendes, I., Gonçalves, M. M., & Neimeyer, R. A. Innovative moments in grief therapy: reconstructing meaning following perinatal death. Death Studies, 2012: 36; 795–818.

Andersson Wretmark, A. The spirit of survival, loss and grief: the issue of surviving twins. MIDIRS Midwifery Digest, 2012: 22; 423–429.

Armstrong. Exploring fathers’ experiences of pregnancy after a prior perinatal loss. MCN. The American Journal Of Maternal Child Nursing, 2001: 26; 147–153.

Armstrong, D. S. Impact of prior perinatal loss on subsequent pregnancies. Journal Of Obstetric, Gynecologic, And Neonatal Nursing: JOGNN / NAACOG, 2004: 33; 765–773.

Armstrong, D. S. Perinatal loss and parental distress after the birth of a healthy infant. Advances In Neonatal Care: Official Journal Of The National Association Of Neonatal Nurses, 2007: 7; 200–206.

Armstrong, D. S., Hutti, M. H., & Myers, J. The influence of prior perinatal loss on parents’ psychological distress after the birth of a subsequent healthy infant. JOGNN: Journal of Obstetric, Gynecologic & Neonatal Nursing, 2009: 38; 654–666.

Avelin, P., Erlandsson, K., Hildingsson, I., & Rådestad, I. Swedish parents’ experiences of parenthood and the need for support to siblings when a baby is stillborn. Birth (Berkeley, Calif.), 2011: 38; 150–158.

Avelin, P., Gyllenswärd, G., Erlandsson, K., & Rådestad, I. Adolescents’ Experiences of Having a Stillborn Half-Sibling. Death Studies, 2014: 38; 557–562.

Avelin, P., Rådestad, I., Säflund, K., Wredling, R., & Erlandsson, K. Parental grief and relationships after the loss of a stillborn baby. Midwifery, 2013: 29; 668–673.

Barr, P. Relation between grief and subsequent pregnancy status 13 months after perinatal bereavement. Journal of Perinatal Medicine, 2006: 34; 207–211.

Barr, P. Negative self-conscious emotion and grief: an actor-partner analysis in couples bereaved by stillbirth or neonatal death. Psychology And Psychotherapy, 2012: 85; 310–326.

Barr, P.& Cacciatore, J. Problematic emotions and maternal grief. Omega: Journal of Death & Dying, 2007: 56; 331–348.

Barr, P., & Cacciatore, J. Personal Fear of Death and Grief in Bereaved Mothers. Death Studies, 2008: 32; 445–460.

Bernazzani, O., & Bifulco, A. Motherhood as a vulnerability factor in major depression: the role of negative pregnancy experiences. Social Science & Medicine, 2003: 56; 1249–1260.

Blackmore, E. R., Côté-Arsenault, D., Tang, W., Glover, V., Evans, J., Golding, J., & O’Connor, T. G. Previous prenatal loss as a predictor of perinatal depression and anxiety. The British Journal Of Psychiatry: The Journal Of Mental Science, 2011: 198; 373–378.

Breines Tone, S., Wahl Klopstad, A., Vangen, S., & Eberhard-Gran, M. [Do previous abortions cause fear of childbirth?]. Tidsskrift for Den Norske Lægeforening : Tidsskrift for Praktisk Medicin, Ny Række, 2013: 133; 635.

Brierley-Jones, L., Crawley, R., Lomax, S., & Ayers, S. Stillbirth and stigma: the spoiling and repair of multiple social identities. Omega, 2015: 70; 143–168.

Cacciatore, J. The unique experiences of women and their families after the death of a baby. Social Work in Health Care, 2010: 49; 134–148.

Cacciatore, J., & Bushfield, S. Stillbirth: the mother’s experience and implications for improving care. Journal of Social Work in End-of-Life & Palliative Care, 2007: 3; 59–79.

Cacciatore, J., DeFrain, J., Jones, K. L. C., & Jones, H. Stillbirth and the couple: a gender-based exploration. Journal of Family Social Work, 2008: 11; 351–370.

Cacciatore, J., Erlandsson, K., & Rådestad, I. Fatherhood and suffering: a qualitative exploration of Swedish men’s experiences of care after the death of a baby. International Journal Of Nursing Studies, 2013: 50; 664–670.

Cacciatore, J., Rådestad, I., & Frederik Frøen, J. Effects of contact with stillborn babies on maternal anxiety and depression. Birth: Issues in Perinatal Care, 2008: 35; 313–320.

Cacciatore, J., Schnebly, S., & Frederik Frøen, J. The effects of social support on maternal anxiety and depression after stillbirth. Health & Social Care in the Community, 2009: 17; 167-76.

Capitulo, K. L., Ramirez, M., Grigoroff-Aponte, B., & Vahey, D. C. Psychometric testing of the new Spanish Short Version of the Perinatal Grief Scale to measure perinatal grief in Spanish-speaking parents. Hispanic Health Care International, 2010: 8; 125–135.

Christiansen, D. M., Elklit, A., & Olff, M. Parents bereaved by infant death: PTSD symptoms up to 18 years after the loss. General Hospital Psychiatry, 2013: 35; 605–611.

Christiansen, D. M., Olff, M., & Elklit, A. Parents bereaved by infant death: sex differences and moderation in PTSD, attachment, coping and social support. General Hospital Psychiatry, 2014: 36; 655–661.

Coleman, P. K., Maxey, C. D., Rue, V. M., & Coyle, C. T. Associations between voluntary and involuntary forms of perinatal loss and child maltreatment among low-income mothers. Acta Paediatrica (Oslo, Norway: 1992), 2005: 94; 1476–1483.

Côté-Arsenault, D. The influence of perinatal loss on anxiety in multigravidas. Journal Of Obstetric, Gynecologic, And Neonatal Nursing: JOGNN / NAACOG, 2003: 32; 623–629.

Côté-Arsenault, D. Threat appraisal, coping, and emotions across pregnancy subsequent to perinatal loss. Nursing Research, 2007: 56; 108–116.

Cote-Arsenault, D., & Donato, K. Emotional cushioning in pregnancy after perinatal loss. Journal of Reproductive & Infant Psychology, 2011: 29; 81–92.

Côté-Arsenault, D., & Donato, K. L. Restrained expectations in late pregnancy following loss. Journal Of Obstetric, Gynecologic, And Neonatal Nursing: JOGNN / NAACOG, 2007: 36; 550–557.

Côté-Arsenault, D., Donato, K. L., & Earl, S. S. Watching & worrying: early pregnancy after loss experiences. MCN. The American Journal Of Maternal Child Nursing, 2006: 31; 356–363.

Côté-Arsenault, D., & Freije, M. M. Support groups helping women through pregnancies after loss. Western Journal Of Nursing Research, 2004: 26; 650–670.

Cote-Arsenault, D., & Marshall, R. One foot in-one foot out: weathering the storm of pregnancy after perinatal loss. Research In Nursing & Health, 2000: 23; 473–485.

Couto, E. R., Couto, E., Vian, B., Gregório, Z., Nomura, M. L., Zaccaria, R., & Passini Jr, R. Quality of life, depression and anxiety among pregnant women with previous adverse pregnancy outcomes. São Paulo Medical Journal = Revista Paulista De Medicina, 2009: 127; 185–189.

Cowchock, F. S., Lasker, J. N., Toedter, L. J., Skumanich, S. A., & Koenig, H. G. Religious beliefs affect grieving after pregnancy loss. Journal Of Religion And Health, 2010: 49; 485–497.

Doug, C., & Carrick-Sen, D. Prevalence of Fathers experience of PTSD following Childbirth. Journal of Reproductive and Infant Psychology, 2010: 28; e4–e5.

Downe, S., Schmidt, E., Kingdon, C., & Heazell, A. E. P. Bereaved parents’ experience of stillbirth in UK hospitals: a qualitative interview study. BMJ Open, 2013: 3.

Dyregrov, A., & Gjestad, R. Sexuality following the loss of a child. Death Studies, 2011: 35; 289–315.

Erlandsson, K., Avelin, P., Säflund, K., Wredling, R., & Rådestad, I. Siblings’ farewell to a stillborn sister or brother and parents' support to their older children: a questionnaire study from the parents' perspective. Journal Of Child Health Care: For Professionals Working With Children In The Hospital And Community, 2010: 14; 151–160.

Erlandsson, K., Lindgren, H., Malm, M.-C., Davidsson-Bremborg, A., & Rådestad, I. Mothers’ experiences of the time after the diagnosis of an intrauterine death until the induction of the delivery: a qualitative Internet-based study. The Journal Of Obstetrics And Gynaecology Research, 2011: 37; 1677–1684.

Erlandsson, K., Saflund, K., Wredling, R., & Rådestad, I. Support After Stillbirth and Its Effect on Parental Grief Over Time. Journal of Social Work in End-of-Life & Palliative Care, 2011: 7; 139–152.

Erlandsson, K., Warland, J., Cacciatore, J., & Rådestad, I. Seeing and holding a stillborn baby: mothers’ feelings in relation to how their babies were presented to them after birth--findings from an online questionnaire. Midwifery, 2013: 29; 246–250.

Fenstermacher, K. H. Enduring to gain new perspective: a grounded theory study of the experience of perinatal bereavement in Black adolescents. Research In Nursing & Health, 2014: 37; 135–143.

Forhan, M. Doing, being, and becoming: a family’s journey through perinatal loss. The American Journal Of Occupational Therapy: Official Publication Of The American Occupational Therapy Association, 2010: 64; 142–151.

Fottrell, E., Kanhonou, L., Goufodji, S., Béhague, D. P., Marshall, T., Patel, V., & Filippi, V. Risk of psychological distress following severe obstetric complications in Benin: the role of economics, physical health and spousal abuse. The British Journal Of Psychiatry: The Journal Of Mental Science, 2010: 196; 18–25.

Franche, R. L. Psychologic and obstetric predictors of couples’ grief during pregnancy after miscarriage or perinatal death. Obstetrics And Gynecology, 2001: 97; 597–602.

Frøen, J. F., Cacciatore, J., McClure, E. M., Kuti, O., Jokhio, A. H., Islam, M., & Shiffman, J. Stillbirths: why they matter. Lancet, 2011: 377; 1353–1366.

Gaudet, C. Pregnancy after perinatal loss: association of grief, anxiety and attachment. Journal of Reproductive & Infant Psychology, 2010: 28; 240–251.

Gausia, K., Moran, A. C., Ali, M., Ryder, D., Fisher, C., & Koblinsky, M. Psychological and social consequences among mothers suffering from perinatal loss: perspective from a low income country. BMC Public Health, 2011: 11, 451.

Gold J, K., & Johnson, T. R. B. Mothers at risk: maternal mental health outcomes after perinatal death. Obstetrics & Gynecology, 2014: 123, 6S–6S.

Gold, K. J., Boggs, M. E., Mugisha, E., & Palladino, C. L. Internet message boards for pregnancy loss: who’s on-line and why? Women’s Health Issues: Official Publication Of The Jacobs Institute Of Women's Health, 2012: 22; e67–e72.

Gold, K. J., Boggs, M. E., Muzik, M., & Sen, A. Anxiety disorders and obsessive compulsive disorder 9 months after perinatal loss. General Hospital Psychiatry, 2014: 36; 650–654.

Gold, K. J., Sen, A., & Hayward, R. A. Marriage and cohabitation outcomes after pregnancy loss. Pediatrics, 2010: 125; e1202–e1207.

Gravensteen, I. K., Helgadottir, L. B., Jacobsen, E.-M., Sandset, P. M., & Ekeberg, Ø. Long-term impact of intrauterine fetal death on quality of life and depression: a case-control study. BMC Pregnancy And Childbirth, 2012: 12, 43.

Grout, L. A., & Romanoff, B. D. The myth of the replacement child: parents’ stories and practices after perinatal death. Death Studies, 2000: 24; 93–113.

Hazen, M. A. Societal and workplace responses to perinatal loss: disenfranchised grief or healing connection. Human Relations, 2003: 56; 147–166.

Heazell, A. E. P., McLaughlin, M.-J., Schmidt, E. B., Cox, P., Flenady, V., Khong, T. Y., & Downe, S. A difficult conversation? The views and experiences of parents and professionals on the consent process for perinatal postmortem after stillbirth. BJOG: An International Journal Of Obstetrics And Gynaecology, 2012: 119; 987–997.

Hiruta, A. The existence of the deceased children in the grieving process of mothers who have experienced stillbirth [Japanese]. Journal of Japan Academy of Midwifery, 2009: 23; 59–71.

Hogue, C. J. R., Parker, C. B., Willinger, M., Temple, J. R., Bann, C. M., Silver, R. M., … Goldenberg, R. L. The association of stillbirth with depressive symptoms 6-36 months post-delivery. Paediatric And Perinatal Epidemiology, 2015: 29; 131–143.

Hopkins Hutti, M., Armstrong S., D., & Myers, J. Continuing Psychometric Evaluation of the Perinatal Grief Intensity Scale in the Subsequent Pregnancy After Perinatal Loss. JOGNN: Journal of Obstetric, Gynecologic & Neonatal Nursing, 2014: 43; S82–S82.

Hsu, M., Tseng, Y., Banks, J. M., & Kuo, L. Interpretations of stillbirth. Journal of Advanced Nursing, 2004: 47; 408–416.

Hsu, M., Tseng, Y., & Kuo, L. Transforming loss: Taiwanese women’s adaptation to stillbirth. Journal of Advanced Nursing, 2002: 40; 387–395.

Huberty, J. L., Coleman, J., Rolfsmeyer, K., & Wu, S. A qualitative study exploring women’s beliefs about physical activity after stillbirth. BMC Pregnancy And Childbirth, 2014: 14, 26.

Hughes, P., Turton, P., Hopper, E., McGauley, G. A., & Fonagy, P. Disorganised attachment behaviour among infants born subsequent to stillbirth. Journal Of Child Psychology And Psychiatry, And Allied Disciplines, 2001: 42; 791–801.

Hughes, P., Turton, P., McGauley, G. A., & Fonagy, P. Factors that predict infant disorganization in mothers classified as U in pregnancy. Attachment & Human Development, 2006: 8; 113–122.

Hutti H., M., Armstrong S., D., Myers A., J., & Hall A., L. Grief Intensity, Psychological Well-Being, and the Intimate Partner Relationship in the Subsequent Pregnancy after a Perinatal Loss. JOGNN: Journal of Obstetric, Gynecologic & Neonatal Nursing, 2015: 44; 42–50.

Hutti, M. H., Armstrong, D. S., & Myers, J. Healthcare utilization in the pregnancy following a perinatal loss. MCN. The American Journal Of Maternal Child Nursing, 2011: 36; 104–111.

Jaffe, J., & Diamond, O. Grieving a reproductive loss. Reproductive Trauma: Psychotherapy with Infertility and Pregnancy Loss Clients. 2011: 91.

Jind, L. Parents’ adjustment to late abortion, stillbirth or infant death: the role of causal attributions. Scandinavian Journal Of Psychology, 2003: 44; 383–394.

Kavanaugh, K., & Hershberger, P. Perinatal loss in low-income African American parents. Journal Of Obstetric, Gynecologic, And Neonatal Nursing: JOGNN / NAACOG, 2005: 34; 595–605.

Kavanaugh, K., Trier, D., & Korzec, M. Social support following perinatal loss. Journal Of Family Nursing, 2004: 10; 70–92.

Kelley, M. C., & Trinidad, S. B. Silent loss and the clinical encounter: Parents’ and physicians' experiences of stillbirth-a qualitative analysis. BMC Pregnancy And Childbirth, 2012: 12, 137.

Kerslake, K., & Parkinson, S. Learning from loss: remembering Rachel and Thomas. Essentially MIDIRS, 2012: 3; 17–21.

Kersting, A., Kroker, K., Schlicht, S., Baust, K., & Wagner, B. Efficacy of cognitive behavioral internet-based therapy in parents after the loss of a child during pregnancy: pilot data from a randomized controlled trial. Archives Of Women’s Mental Health, 2011: 14; 465–477.

Kitson, C. Fathers experienced stillbirth as a waste of life and needed to protect their partners and express grief in their own way. Evidence Based Nursing, 2002: 5; 61.

Lacasse, J. R., & Cacciatore, J. Prescribing of Psychiatric Medication to Bereaved Parents Following Perinatal/Neonatal Death: An Observational Study. Death Studies, 2014: 38; 589–596.

Lang, A., Fleiszer R., A., Duhamel, F., Sword, W., Gilbert R., K., & Corsini-Munt, S. Perinatal Loss and Parental Grief: The Challenge of Ambiguity and Disenfranchised Grief. Omega: Journal of Death & Dying, 2011: 63; 183–196.

Lang, A., Goulet, C., & Amsel, R. Explanatory model of health in bereaved parents post-fetal/infant death. International Journal Of Nursing Studies, 2004: 41; 869–880.

Lee, B. From grief to new life--the impact of the loss of a baby on future pregnancy. RCM Midwives Journal: Official Journal Of The Royal College Of Midwives, 2002: 5; 190–191.

Lee, C. “She was a person, she was here”: The experience of late pregnancy loss in Australia. Journal of Reproductive & Infant Psychology, 2012: 30; 62–76.

Lindgren, H., Malm, M.-C., & Rådestad, I. You Don’t Leave Your Baby-Mother's Experiences After A Stillbirth. Omega: Journal of Death & Dying, 2013: 68; 337–346.

#168 Łuczak-Wawrzyniak, J., Czarnecka-Iwańczuk, M., Bukowska, A., & Konofalska, N. (2010). [Early and late psychological effects of pregnancy loss]. Ginekologia Polska, 81(5), 374–377.

Malm, M.-C., Rådestad, I., Erlandsson, K., & Lindgren, H. Waiting in no-man’s-land - mothers' experiences before the induction of labour after their baby has died in utero. Sexual & Reproductive Healthcare: Official Journal Of The Swedish Association Of Midwives, 2011; 2; 51–55.

McCreight, B. S. A grief ignored: narratives of pregnancy loss from a male perspective. Sociology Of Health & Illness, 2004: 26; 326–350.

McCreight, B. S. Perinatal loss: a qualitative study in Northern Ireland. Omega: Journal of Death & Dying, 2008: 57; 1–19.

Mehran, P., Simbar, M., Shams, J., Ramezani-Tehrani, F., & Nasiri, N. History of perinatal loss and maternal–fetal attachment behaviors. Women & Birth, 2013: 26; 185–189.

Munk-Olsen, T., Bech, B. H., Vestergaard, M., Li, J., Olsen, J., & Laursen, T. M. Psychiatric disorders following fetal death: a population-based cohort study. BMJ Open, 2014; 4; e005187–e005187.

Murphy Louise, S. Finding the positive in loss: stillbirth and its potential for parental empowerment. Bereavement Care, 2012: 31; 98–103.

Murphy, S. Reclaiming a moral identity: stillbirth, stigma and “moral mothers”. Midwifery, 2012: 28; 476–480.

Newitt, M. Spiritual support. Midwives, 2014: 17; 48–49.

Nordlund, E., Borjesson, A., Cacciatore, J., Pappas, C., Randers, I., & Radestad, I. When a baby dies: Motherhood, psychosocial care and negative affect. British Journal of Midwifery, 2012: 20; 780–784.

O’Leary, J., & Thorwick, C. Fathers’ perspectives during pregnancy, postperinatal loss. Journal Of Obstetric, Gynecologic, And Neonatal Nursing: JOGNN / NAACOG, 2006: 35; 78–86.

O’Leary, J., & Warland, J. Untold Stories of Infant Loss: The Importance of Contact With the Baby for Bereaved Parents. Journal of Family Nursing, 2013: 19; 324–347.

O’Leary, J., Warland, J., & Parker, L. Bereaved parents’ perception of the grandparents' reactions to perinatal loss and the pregnancy that follows. Journal Of Family Nursing, 2011: 17; 330–356.

Obi, S. N., Onah, H. E., & Okafor, I. I. Depression among Nigerian women following pregnancy loss. International Journal Of Gynaecology And Obstetrics: The Official Organ Of The International Federation Of Gynaecology And Obstetrics, 2009: 105; 60–62.

Pantke, R., & Slade, P. Remembered parenting style and psychological well-being in young adults whose parents had experienced early child loss. Psychology And Psychotherapy, 2006; 79; 69–81.

Pector, E. A. How bereaved multiple-birth parents cope with hospitalization, homecoming, disposition for deceased, and attachment to survivors. Journal Of Perinatology: Official Journal Of The California Perinatal Association, 2004: 24; 714–722.

Peel, E. Pregnancy loss in lesbian and bisexual women: an online survey of experiences. Human Reproduction, 2010: 25; 721-7.

Pidgeon, C. Life after stillbirth: a father’s story. RCM Midwives: The Official Journal Of The Royal College Of Midwives, 2007: 10; 288–289.

Price, SK. Stepping back to gain perspective: pregnancy loss history, depression, and parenting capacity in the Early Childhood Longitudinal Study, Birth Cohort (ECLS-B). Death Studies, 2008: 32; 97-122.

Rådestad, I. Stillbirth: care and long-term psychological effects. British Journal of Midwifery, 2001: 9; 474–480.

Rådestad, I., Hutti, M., Säflund, K., Onelöv, E., & Wredling, R.. Advice given by health-care professionals to mothers concerning subsequent pregnancy after stillbirth. Acta Obstetricia Et Gynecologica Scandinavica, 2010: 89; 1084–1086.

Rådestad, I., Malm, M.-C., Lindgren, H., Pettersson, K., & Larsson, L.-L. F. Being alone in silence - mothers’ experiences upon confirmation of their baby's death in utero. Midwifery, 2014: 30; e91–e95.

Radestad, I., Saflund, K., Wredling, R., Onelöv, E., & Steineck, G. Holding a stillborn baby: mothers’ feelings of tenderness and grief. British Journal of Midwifery, 2009: 17; 178–180.

Rådestad, I., Surkan, P. J., Steineck, G., Cnattingius, S., Onelöv, E., & Dickman, P. W. Long-term outcomes for mothers who have or have not held their stillborn baby. Midwifery, 2009: 25; 422–429.

Reid, M. The loss of a baby and the birth of the next infant: The mother’s experience. Journal of Child Psychotherapy, 2007: 33; 181.

Ryninks, K., Roberts-Collins, C., McKenzie-McHarg, K., & Horsch, A. Mothers’ experience of their contact with their stillborn infant: an interpretative phenomenological analysis. BMC Pregnancy And Childbirth, 2014: 14, 203.

Säflund, K., Sjögren, B., & Wredling, R. The role of caregivers after a stillbirth: views and experiences of parents. Birth (Berkeley, Calif.), 2004: 31; 132–137.

Säflund, K., & Wredling, R. Differences within couples’ experience of their hospital care and well-being three months after experiencing a stillbirth. Acta Obstetricia Et Gynecologica Scandinavica, 2006: 85; 1193–1199.

Sanchez, N. A. Mothers’ perceptions of benefits of perinatal loss support offered at a major university hospital. Journal of Perinatal Education, 2001: 10; 23–30.

Sansoni, J., & Giaquinto, A. Grief of parents for a pre-born child loss [Italian]. Professioni Infermieristiche, 2001: 54; 3–18.

Schiff, M. A., & Grossman, D. C. Adverse perinatal outcomes and risk for postpartum suicide attempt in Washington state, 1987-2001. Pediatrics, 2006: 118; e669–e675.

Schreiber, H. Parents experiencing a perinatal death found that their interactions with social institutions devalued their loss [commentary on Malacrida C. Complicating mourning: the social economy of perinatal death. QUAL HEALTH RES 1999 Jul;9(4):504-19]. Evidence Based Nursing, 2000: 3; 27.

Shreffler, M., Greil, L., & McQuillan, J. Pregnancy loss and distress among U.S. women. Family Relations: An Interdisciplinary Journal of Applied Family Studies, 2011: 60; 342.

Sisay Molla, M., Yirgu, R., Gobezayehu Gebremariam, A., & Sibley M., L. A Qualitative Study of Attitudes and Values Surrounding Stillbirth and Neonatal Mortality Among Grandmothers, Mothers, and Unmarried Girls in Rural Amhara and Oromiya Regions, Ethiopia: Unheard Souls in the Backyard. Journal of Midwifery & Women’s Health, 2014: 59, S110–7.

St John, A., Cooke, M., & Goopy, S. Shrouds of silence: three women’s stories of prenatal loss. The Australian Journal Of Advanced Nursing: A Quarterly Publication Of The Royal Australian Nursing Federation, 2006: 23; 8–12.

Sun, H.-L., Sinclair, M., Kernohan, G. W., Chang, T.-H., & Patterson, H. Sailing against the tide: Taiwanese women’s journey from pregnancy loss to motherhood. MCN. The American Journal Of Maternal Child Nursing, 2011: 36; 127–133.

Sun, J.-C., Rei, W., & Sheu, S.-J. Seeing or not seeing: Taiwan’s parents' experiences during stillbirth. International Journal Of Nursing Studies, 2014: 51; 1153–1159.

Surkan, P. J., Rådestad, I., Cnattingius, S., Steineck, G., & Dickman, P. W. Events after stillbirth in relation to maternal depressive symptoms: a brief report. Birth (Berkeley, Calif.), 2008: 35; 153–157.

Surkan, P. J., Rådestad, I., Cnattingius, S., Steineck, G., & Dickman, P. W. Social support after stillbirth for prevention of maternal depression. Acta Obstetricia et Gynecologica Scandinavica, 2009:88; 1358-64.

Sutan, R., Amin, R. M., Ariffin, K. B., Teng, T. Z., Kamal, M. F., & Rusli, R. Z. Psychosocial impact of mothers with perinatal loss and its contributing factors: an insight. Journal Of Zhejiang University. Science. B, 2010: 11; 209–217.

Sutan, R., & Miskam, H. M. Psychosocial impact of perinatal loss among Muslim women. BMC Women’s Health, 2012: 12, 15.

Swanson, P. B., Pearsall-Jones, J. G., & Hay, D. A. (2002). How mothers cope with the death of a twin or higher multiple. Twin Research: The Official Journal Of The International Society For Twin Studies, 2002: 5; 156–164.

Takaki, J., & Hibino, Y. Family-related opinions and stressful situations associated with psychological distress in women undergoing infertility treatment. International Journal Of Environmental Research And Public Health, 2014: 11; 9068–9081.

Tran, D. T., Roberts, C. L., Jorm, L. R., Seeho, S., & Havard, A. Change in smoking status during two consecutive pregnancies: a population-based cohort study. BJOG: An International Journal Of Obstetrics And Gynaecology, 2014: 121; 1611–1620.

Tseng, Y.-F., Chen, C.-H., & Wang, H.-H. Taiwanese women’s process of recovery from stillbirth: a qualitative descriptive study. Research In Nursing & Health, 2014: 37; 219–228.

Turton, P., Badenhorst, W., Hughes, P., Ward, J., Riches, S., & White, S. Psychological impact of stillbirth on fathers in the subsequent pregnancy and puerperium. The British Journal Of Psychiatry: The Journal Of Mental Science, 2006:188; 165–172.

Turton, P., Badenhorst, W., Pawlby, S., White, S., & Hughes, P. Psychological vulnerability in children next-born after stillbirth: a case-control follow-up study. Journal Of Child Psychology And Psychiatry, And Allied Disciplines, 2009; 50; 1451–1458.

Turton, P., Evans, C., & Hughes, P. Long-term psychosocial sequelae of stillbirth: phase II of a nested case-control cohort study. Archives Of Women’s Mental Health, 2009: 12; 35–41.

Turton, P., Hughes, P., Fonagy, P., & Fainman, D. An investigation into the possible overlap between PTSD and unresolved responses following stillbirth: an absence of linkage with only unresolved status predicting infant disorganization. Attachment & Human Development, 2004: 6; 241–253.

Van der Sijpt, E. The unfortunate sufferer: discursive dynamics around pregnancy loss in Cameroon. Medical Anthropology, 2014: 33; 395–410.

Van, P. Breaking the silence of African American women: healing after pregnancy loss. Health Care For Women International, 2001: 22; 229–243.

Van, P., & Meleis, A. I. Coping with grief after involuntary pregnancy loss: perspectives of African American women. Journal Of Obstetric, Gynecologic, And Neonatal Nursing: JOGNN / NAACOG, 2003: 32; 28–39.

Vance, J. C., Boyle, F. M., Najman, J. M., & Thearle, M. J. Couple distress after sudden infant or perinatal death: a 30-month follow up. Journal Of Paediatrics And Child Health, 2002: 38; 368–372.

Varney, S. Perinatal loss and its vicissitudes. Journal of Infant, Child & Adolescent Psychotherapy, 2014: 13; 51.

Vidal, M. [Pregnancy after perinatal death: concerning the relationship of mother with the survivor baby]. Ciência & Saúde Coletiva, 2010: 15; 3185–3190.

Warland, J., O’Leary, J., McCutcheon, H., & Williamson, V. Parenting paradox: parenting after infant loss. Midwifery, 2011: 27; e163–e169.

Welborn, J. M. The experience of expressing and donating breast milk following a perinatal loss. Journal Of Human Lactation: Official Journal Of International Lactation Consultant Association, 2012: 28; 506–510.

Wilson, R. E. Parents’ support of their other children after a miscarriage or perinatal death. Early Human Development, 2001: 61; 55–65.

Wood, L., & Quenby, S. Women’s perceptions of stressful life events in relation to pre-term birth. British Journal of Midwifery, 2011: 19; 107–114.

Yamazaki, A. Living with stillborn babies as family members: Japanese women who experienced intrauterine fetal death after 28 weeks gestation. Health Care For Women International, 2010: 31; 921–937.

**Table 1** – Thematic sentences, frequency effect sizes (FES) and quotes from extracted findings in the metasummary

| **Thematic sentence** | **Frequency Effect Sizes -**  **FES (%)** | **Quotes from extracted findings** | **References** |
| --- | --- | --- | --- |
| Stillbirth has been associated with a number of emotional, depressive and other negative psychological symptoms – (Negative psychological symptoms) | 77.1 | ‘When they showed me there was no heartbeat I was gutted. It was like someone had ripped out my heart, my heart broke and it's never gone back right. I couldn't go out of the house. I just couldn't cope with it all and I didn't eat for weeks, I just wanted to die, badly’ ^McCreight 2008^  ‘I don’t deserve to feel happiness, and I should suffer as well.’ ^Hiruta 2009^  ‘I started persecuting myself harshly, thinking that it was me who let him/her die’ ^Hiruta 2009^  ‘a week or so after (he) died…I wanted to die. I never tried. I just wished I wasn't here*.’* ^Cacciatore 2010^  ‘I have moments when I apologize for killing our daughter even though there was nothing I could do to stop it.’ ^Cacciatore 2010^  *‘* Yes, depression, but more anger— anger at the medical system, and friends, family, and ourselves.’ ^Kelley 2012^  ‘I am depressed, saddened, hurt, empty, guilt and lonely. I cry every day. I will mourn him forever ‘ ^Lee 2012^ | Huberty 2014, Sun 2014, Gold 2014, Rynlinks 2014, Gravensteen 2012, Sutan 2012, Avelin 2011, Cacciatore 2007, Pidgeon 2007, Surkan 2008, Cacciatore 2009, Turton 2006, Sisay 2014, Kavanaugh 2004, Sutan 2010, Turton 2006, Turton 2009, Fottrell 2010, Kelley 2012, Gausia 2011, Kersting 2011, Adeyemi 2008, Cacciatore 2008, O’Leary 2006, Saflund 2004, Barr 2007, Tseng 2014, Avelin 2013, Armstrong 2001, Barr 2012, Lingren 2013, Fenstermacher 2014, Radestad 2014, O’Leary 2013, Hughes 2006, Murphy 2012, Anderson 2012, Hopkins Hutti 2014, Lee 2012,Dyregrov 2011, Doug 2010, Hiruta 2009, Sun 2011, Capitulo 2010, O’Leary 2006, Vance 2012, Radestad 2001, Cacciatore 2013, Peel 2010, Gold 2012, Blackmore 2011, Cowchock 2010, Radestad 2009, Hsu 2004, McCreight 2004, Pector 2004, Schiff 2006, Kavanaugh 2005, St John 2006, Kitson 2002, Hsu 2002, Christiansen 2013,Breines Simonsen 2013, Luczak Wawrzyniak 2010, Alves 2012, Kertsing 2011, Aho 2011, Yamazaki 2010, Surkan 2009, Saflund 2006, Nordlund 2012, Cacciatore 2010, Cacciatore 2008, Eriandson 2011, Kavanaugh 2004, Swanson 2002, Price 2008, McCreight 2008, Armstrong 2007, Cacciatore 2007, Barr 2006, Takaki 2014, Kerslake 2012, Couto 2009, Jind 2003, Van 2003, Lee 2002, Van 2001, Wilson 2001, Bernazzani 2003, Varney 2014, Jaffe 2011, Reid 2007, Hogue 2015, Gold 2014, Lacasse 2014, Newitt 2014, Brierley Jones 2015, Munk-Olsen 2014, Hutti 2015, Forhan 2010 Malm 2011, Radsted 2009, Avelin 2014, Obi 2009, Turton 2004, Murphy 2012, Gravensteen 2012, Cote-Arsenault 2000, Christiansen 2013 |
| Parental grief following stillbirth may not legitimised by health professionals, family and society -(Disenfranchised grief) | 31.2 | ’She told my neighbour that I am “an infertile hen who eats a lot but does not carry any eggs” I felt so upset. I felt that I was devalued as a ‘bad daughter-in-law’ just because I could not give birth to an heir. ‘I hope that I can have a baby as soon as possible. So I can end this nightmare.’ ^Hsu 2002^  ‘Because my child is not within my reach, there is a reality that I am not acknowledged as a mother.’ ^Hiruta 2009^  ‘When asked who supported them, three fathers simply replied, “ No one.” ^O’Leary 2006^  ‘When I told people I lost my daughter, people assumed that my daughter died after birth, so they started asking her age. When I told them that she died during pregnancy, days before her due date, they responded with a deflating “Oh.” That word “Oh” made it sound as though death before birth is not something to be upset about.’ ^Hiruta 2009^  ‘People didn't know what to say. Many treated me like a leper and still do.’ ^Brierley-Jones 2015^ | Hsu 2002, Sutan 2012, Avelin 2011, Schreffler 2011, Sun 2014, Sisay 2014, Kavanaugh 2004, Kelley 2012, Gausia 2011, O’Leary 2006, Tseng 2014, Lang 2011, O’Leary 2013, Murphy 2012, Anderson 2012, Cote-Arsenault 2004, Hiruta 2009, Sun 2011, Blackmore 2011, Peel 2010, Cacciatore 2013,Cote-Arsenault 2011, Sanchez 2001, St John 2006, Yamazaki 2010, Nordlund 2012, Cacciatore 2010, Cacciatore 2008, Eriandson 2011, Kavanaugh 2004, Swanson 2002, McCreight 2008, Cacciatore 2007, Downe 2013, Kerslake 2012, Van 2003, Wilson 2001, Froen 2011, Jaffe 2011, Newitt 2014, Brierley-Jones 2015, Avelin 2014, O’Leary 2011, Schreiber 2000, Van der Sijpt 2014 |
| A stillbirth may have a positive or negative impact on relationships, for example, through incongruent grief reactions - (Effect on relationships/incongruent grief) | 28.5 | ‘Initially my husband 'shielded' me from everything and everyone. After a very short time he began refusing to acknowledge baby had existed and this put a great strain on us both. We eventually divorced.’ ^Cacciatore 2008^  ‘Our relationship as a couple has changed in that there's an unspoken bond, as if I know him on a different level than I did before.’ ^Cacciatore 2008^  ‘He refused to discuss our child, saying it was in the past… He was less loving and just wanted sex instead of an act of love. I felt less attractive to him and withdrew as sex became more violent... I later found out he was having affairs.‘ ^Cacciatore 2008^ | Turton 2006, Turton 2009, Fottrell 2010, Gausia 2011, Yamazaki 2010, Gold 2010, Tseng 2014, Avelin 2013, Armstrong 2001, Lang 2011, Warland 2011, Cote-Arsenault 2004, Hopkins Hutti 2014, Dyregrov 2011, Doug 2010, Sun 2011, Wood 2011, Capitulo 2010, Vance 2002, Radestad 2001, Kavanaugh 2005, Hsu 2002, Yamazaki 2010, Surkan 2009, Saflund 2006, Cacciatore 2008, Kavanaugh 2004, Swanson 2002, Armstrong 2007, Downe 2013. Kerslake 2012, Jind 2003, Varney 2014, Jaffe 2011, Reid 2007, Brierly-Jones 2015, Christiansen 2014, Eriandsson 2011, Lang 2004, Murphy 2012, Cote-Arsenault 2000 |
| In subsequent pregnancy some parents may feel isolated and outside the boundaries of normality, and have a number of emotional responses including depressive and other psychological symptoms – (Negative psychological symptoms in subsequent pregnancies) | 27.1 | **‘**You'd have to be like brain dead to go through a pregnancy after losing a baby and be able to take it easy. Am I confident? No. Will I relax? No. There is not a point that I will relax until they are out and breathing and hearts beating.’ ^Cote-Arsenault 2004^  ‘Yes, I felt as though I could not emotionally attach myself to this baby inside me until actually I held him.’ ^Cote-Arsenault 2011^  'You're kind of in this position of fresh grief...but you're also dealing with this new pregnancy, and there's not really room to talk about that.’ ^Cote-Arsenault 2004^ | Sutan 2012, Armstrong 2009, Turton 2006, Sutan 2010, Turton 2009, Adeyemi 2008, O’Leary 2006, Armstrong 2004, Armstrong 2001, Mehran 2013, Lang 2011, O’Leary 2013, Warland 2011, Cote-Arsenault 2004, Hopkins Hutti 2014, Hughes 2001, Sun 2011, Wood 2011, Cote-Arsenault 2011, Gaudet 2010, Vance 2002, Cote-Arsenault 2006, Blackmore 2011,Cote-Arsenault 2007, St John 2006, Cote-Arsenault 2003, Vidal 2010, Breines 2013, Swanson 2002, Armstrong 2007, Van 2003, Lee 2002, Varney 2014, Jaffe 2011, Gold 2014, Brierley-Jones 2015, Hutti 2015, O’Leary 2011, Cote-Arsenault 2000 |
| Stillbirth may change parents’ approach to life and death, self-esteem, personal identity, and sense of control in the subsequent pregnancy, parenthood and childrearing period – (Approach to life & death, self-esteem, own identity) | 25.7 | ‘Our daughter’s death transformed us in many ways—spiritually, emotionally, our perspective on life, and our careers as well. We are not the same people.’ ^Swanson 2002^  ”‘I've definitely learned to live in the present and know that life can turn on a dime, at any old time.’ ^Cote Arsenault 2004^  ‘I am a mother and I know I can’t be a mom.’ ^Welborn 2012^  ‘I feel like things are much less certain and controllable now. I couldn't save my baby*.’* ^Cacciatore 2008^  ‘We try harder not to sweat the small stuff...we try to just really appreciate each other more...appreciate life more. ‘ ^Cacciatore 2008^  ‘My child's death has changed me to be a more sensitive person to other's feelings. Her death has made me realize not to take things for granted such as my living children.’ ^Cacciatore 2008^  ‘My loss is part of me, of who I am, and makes up my life story*.*’ ^Cacciatore 2007^  ‘I lost my confidence in being a parent. I really question my ability as a mother and second guess my judgment all the time.’ ^Cacciatore 2007^ | Cacciatore 2007, Yamazaki 2010, Saflund 2004, Welborn 2012, Armstrong 2001, Lindgren 2013, Lang 2011, Gold 2014, Sisay 2014, Murphy 2012, Warland 2011, Barr 2008, Lee 2012, Dyregov 2011, Hiruta 2009, Sun 2011, Radestad 2009, Schiff 2006, Peel 2010, Hsu 2004, McCreight 2004, Kavanaugh 2005, Avelin 2014, Hsu 2002, Nordlund 2012, Cacciatore 2010, Cacciatore 2008, Eriandson 2011, Swanson 2002, Cacciatore 2008, Downe 2013, Van 2001, Varney 2014, Jaffe 2011, Newitt 2014, Grout 2000, Malm 2011, Cote-Arsenault 2004 |
| Stillbirth can have an adverse impact on siblings, including the surviving twin and subsequent children – (Impact on siblings/subsequent children) | 23.6 | ‘I am not saying I didn't love my kids, but I was scared to invest everything in them and I could see that I would sometimes force myself to play with Sarah because I knew I was supposed to do that. I think you try and protect yourself.’ ^Warland 2011^  ‘It was very disturbing to have left a dead baby at the hospital with his image in death etched on my mind, and to have a baby at home with the identical face.’ ^Pector 2004^  She (Mary) then went on to say she was aware of emotionally 'holding aloof' because she was afraid to invest in her subsequent child lest he die. She 'never wanted to go through that [pain] again.’ ^Warland 2011^ | Huberty 2014, Sutan 2012, Avelin 2011, Pidgeon 2007, Armstrong 2009, Aho 2011, Armstrong 2004, Turton 2009, Mehran 2013, Eriandson 2010, Anderson 2012, Warland 2011, Hughes 2006, Hiruta 2009, Pector 2004, St John 2006, Vidal 2010, Alves 2012, Radestad 2010, Cacciatore 2010, Cacciatore 2008, Swanson 2002, Price 2008, Hughes 2001, Pantke 2006, Coleman 2005, Van 2003, Lee 202, Wilson 2001, Varney 2014, Reid 2007, Avelin 2014, Turton 2004, Cote-Arsenault 2000 |
| After stillbirth, some parents may alter their activities as a coping strategy including; seeking therapeutic isolation (needing time to themselves), increased or decreased religious activity, increased or decreased sexual activity, and increased engagement with health promoting activities, work and social media. This may all continue into subsequent pregnancies*. -* (Therapeutic activities) | 20.1 | ‘It wasn’t about the physical activity it was about kind of trying to get away.’ ^Huberty 2014^  ‘I was determined not to conceive until I was a non-smoker...I think that was a control thing as well as a risk reduction. I thought I could have some control.’ ^Gold 2014^  ‘I’m pretty much a workaholic. It’s what I do when I get depressed. I busy myself.’ ^O’Leary 2006^  ‘Because sometimes you just have those days where you don't feel like getting out of your pyjamas and those are usually the days when the ladies on the forum help me get moving and get on with life. I cry when I talk to a real person so it was easier to talk to someone online, less emotional.’ ^Gold 2012^  ‘I feel my church family is more than just a group of people. My husband has become less spiritual and focuses on his career*.’* ^Cacciatore 2008^ | Huberty 2014, Sutan 2012, O’Leary 2006, Fenstermacher 2014, Anderson 2012, Warland 2011, Lee 2012, Dyregrov 2011, Sun 2011, Cote- Arsenault 2006, Gold 2012, Cowchock 2010, Kavanaugh 2005, St John 2006, Alves 2012, Cacciatore 2008, Eriandson 2011, Price 2008, Cacciatore 2007, Jind 2003, Van 2003, Lee 2002, Van 2001, Jaffe 2011, Reid 2007, Eriandsson 2011, Avelin 2014, Murphy 2012, Cote-Arsenault 2000 |
| Some parents feel the need to suppress outward grief, including during subsequent pregnancy – (Grief suppression) | 18.1 | ‘I had to be strong for Kate [partner], I had to let her cry on me and then I would get into the car and drive up into the hills and cry to myself. I was trying to support her even though I felt like my whole life had just caved in, you know, my whole life just ended there and then.’ ^McCreight 2004^  ‘I think I genuinely suppressed a lot of my anxiety because of my [desire to protect my] family.’ ‘Yes, I wanted to stay strong for my husband and myself. Outward I was strong but inside I was a mess.’ ^Cote-Arsenault 2011^  ‘I don’t know what to do sometimes. I don’t tell her how I feel. Every morning at 4 AM I’m awake. I don’t tell her that I’ve been up [at night worrying].’ ^O’Leary 2006^  ‘In a few weeks you are supposed to pick yourself up and just keep going as though nothing has happened…your feelings and emotions – they just have to stay hidden all the time.’ ^St John 2006^ | Sutan 2012, Turton 2006, Kelley 2012, O’Leary 2006, Tseng 2014, Armstrong 2001, O’Leary 2013, Anderson 2012, Hiruta 2009, Sun 2011, Cote-Arsenault 2011, Hsu 2004, McCreight 2004, Kavanaugh 2005, St John 2006, Kitson 2002, Cacciatore 2008, Eriandson 2011, Swanson 2002, Barr 2006, Downe 2013, Kerslake 2012, Van 2003, Wilson 2001, Jaffe 2011, Brierley-Jones 2015 |
| Stillbirth may lead to avoidance of activities that remind parents of the pregnancy – (Avoidance of memories) | 13.2 | ‘Some women could not face exercise postnatal as it reminded them of antenatal classes and the baby – ‘I tried doing yoga a few times but I had such a connection with yoga and with [child ’s name] ’s pregnancy … I emotionally could not do it**.’** ^Hubery 2014^ | Huberty 2014, Yamazaki 2010, Hiruta 2009, Pector 2004, Kavanaugh 2005, Nordlund 2012, Cacciatore 2008, Kavanaugh 2004, Kerslake 2012, Van 2003, Varney 2014, Brierley-Jones 2015, Malm 2011, Cote-Arsenault 2000, Lang 2004, O’Leary 2006, Fenstermacher 2014, Anderson 2012, Dyregrov 2011 |
| Women report stigmatisation, rejection, and spousal abuse after stillbirth – (Stigmatisation) | 13.2 | ‘They [hospital staff] just ignored us totally, we were a bit like a bad smell in the ward. Yes, it’s true to a certain extent that men are ignored, you feel you’re almost the bastard that caused all this.’ ^McCreight 2004^  ‘……..you are some kind of freak…...’ ^St John 2006^  ‘Every time I walked into the living room, my in-laws lowered their voices. Mostly, they stopped talking. I disappointed them because I didn’t give them a descendent like every daughter-in-law should do. I felt unwomanly, since I failed to have a baby.’ ^Hsu 2004^  ‘Many members of our family do not talk to us.’ ^Murphy 2012^  ‘The younger women stayed away from me because, you know, I felt like I was bad luck then, you know, when I went to the temple they didn't talk to me, The older women did [talk to me] and um but yeah, they made you feel like, you know, I'd been cursed or something.’ ^Murphy 2012^  ‘A woman who loses her neonate has to start work immediately and she is referred [to] as ‘yewesha aras’ in Amharic, which means that she is like a mother dog who walks around immediately after giving birth.’ ^Sisay 2014^  ‘A woman who loses her babies repeatedly is stigmatized. Her neighbours will insult and humiliate her. They will call her woldo-bela [child-killer, in Amharic], meaning a woman who kills her kids with her evil eye.’ ^Sisay 2014^  ‘I had a lady, a neighbour, literally cross the road and went in the other direction, which was very hurtful.’ ^Murphy 2012^ | Sisay 2014, Fottrell 2010, Kelley 2012, Gausia 2011, Murphy 2012, Cote-Arsenault 2004, Hiruta 2009, Sun 2011, Kavanaugh 2005, Hsu 2002, Nordlund 2012, Kavanaugh 2004, Lee 2002, Murphy 2012, Van der Sijpt, Sun 2014, Lang 2011, McCreight 2004, St John 2006 |
| Parents may have mixed feelings regarding the decisions they made e.g. post mortem or seeing/holding their baby*.* – (Decision making at the time of stillbirth) | 12.5 | ‘I regret that I never had my son naked on my skin. He was wrapped in a blanket when I got him and it is something I miss that I have never felt.’ ^Nordlund 2012^  ‘On reflection, I am glad that I held her. I still remember her weight and the feel of her skin against mine and I carry her photo in my wallet. I think I would be lost without it.’ ^Pidgeon 2007^ | Ryninks 2014, Heazell 2012, Pigeon 2007, Adeyemi 2008, Lee 2012, Hiruta 2009, Radestad 2001, Radestad 2009, Eriandson 2013, Nordlund 2012, Cacciatore 2010, Swanson 2002, McCreight 2008, Cacciatore 2007, Downe 2013, Kerslake 2012, Wilson 2001, Brierley-Jones 2015 |
| Parents may experience external or internal pressures to prioritise or delay conception – (Pressures to delay or prioritise contraception) | 9 | ‘Women did not want to exercise as all they wanted to do was to become pregnant again so did not see the point in getting back in shape again.’ ^Huberty 2014^  ‘After a woman has tried everything, if she continues to lose babies, then she will start a contraceptive in consultation with the husband. My daughter had this problem and now she is using it.’ ^Sisay 2014^ | Huberty 2014, Sisay 2014, Fottrell 2010, Dyregrov 2011, Wood 2011, Kavanaugh 2005, Hsu 2002, Eriandsson 2011, Kavanaugh 2004, Van 2003, Lee 2002, Varney 2014, Jaffe 2011 |
| Bereaved parents may become hypervigilant with siblings and subsequent children, and anxious about other people's children – (Anxiety with siblings and subsequent children) | 7.6 | ‘The cot had only a bottom sheet, he slept uncovered, there was a bare floor, because [carpet attracts dust mites and dust mites might cause SIDS] and there were no soft toys for the same reason.’ ^Warland 2011^ | Warland 2011, Sun 2011, Cote-Arsenault 2006, Pector 2004, Vidal 2010, Cacciatore 2008, Swanson 2002, Pantke 2006, Van 2003, Wilson 2001, O’Leary 2011 |
| Chronic pain and fatigue can follow stillbirth – (Chronic pain and fatigue) | 6.9 | Findings from quantitative studies only | Huberty 2014, Gravensteen 2012, Fottrell 2010, Adeyemi 2008, Avelin 2013, Fenstermacher 2014, Couto 2009, Van 2001, Reid 2007, Lang 2004, |
| Bereaved parents may increase or decrease their use of health care services; and in subsequent pregnancy fathers express a desire to be more included in care – (Use of healthcare services) | 6.9 | ‘During the pregnancy we asked a lot more questions and were a lot more aware generally of the things that could go wrong…I am a lot more challenging of doctors now’ ^Warland 2011^ | Armstrong 2009, Turton 2009, Armstrong 2001, Warland 2011, Cote-Arsenault 2006, Cote-Arsenault 2007, Cote-Arsenault 2003, Hutti 2011, Van 2003, O’Leary 2011 |
| Some parents described parental pride after the birth of their stillborn baby – (Parental pride) | 5.6 | **‘**I felt happy, as I felt I was able to become a mother.’ ^Hiruta 2009^  “He/she was just adorable. My first impression was that he/she was adorable.‘ ^Hiruta 2009^ | Ryninks 2014, Lee 2012, Hiruta 2009, Cacciatore 2013, Nordlund 2012, Downe 2013, Kerslake 2012, Avelin 2014 |
| Potential impact includes employment difficulties and financial debt – (Employment difficulties and financial debt) | 5.6 | ‘I know a girl who was in school and married off by her parents. After the marriage, she repeatedly lost her newborns and was divorced. Not to face the humiliation in the village she ran away to a city and now she is a commercial sex worker.’ ^Sisay 2014^ | Sisay 2014, Fottrell 2010, O’Leary 2006,, Saflund 2006, Cacciatore 2008, Downe 2013, Reid 2007, Gravensteen 2012 |
| Increased substance use has been reported for some parents – (Increased substance use) | 4.2 | Findings from quantitative studies only. | Turton 2006, Adeyemi 2008, O’Leary 2013, Eriandson 2011, Reid 2007, Lacasse 2014 |
| Stillbirth can motivate parents to engage with healthcare improvement including public awareness – (Motivation for engagement in healthcare) | 4.2 | I deal with it in a way that you know, to crusade, to campaign, to make sure things change, to try and take the positives as much as you can out of the whole situation rather than just dwell on, you know, the terrible things that have happened because, you know, you can't change what's happened unfortunately in the past. What you can do is change things for the future so I'm always...glass half full.’ ^Murphy 2012^  I know that I have to go through stuff [like this interview] because I think it's a job that we have to do in a sense to make it real to other people and to widen the understanding of stillbirth.’ ^Murphy 2012^ | Murphy 2012, Warland 2011, McCreight 2004, Yamazaki 2010, Cacciatore 2007, Kerslake 2012, |
| Women may develop a complex emotional response to body image – (Altered body image) | 3.5 | ‘I struggled with low self-esteem for a long time. I think I still cannot relate to my body. I could not stand my body - it had killed my child.’ ^Dyregrov 2011^  ‘I had no child to lay on my breast and felt a strong need to have my husband there, but I also had great difficulties accepting my body because I weighed 10 kilos more than I should and felt very bulky and awful after the birth. In the beginning I could not look at myself, it was very hard to accept my body ‘ ^Dyregrov 2011^ | Huberty 2014, Dyregrov 2011, Eriandson 2011, Lee 2002, Malm 2011 |
| Stillbirth has an adverse affect on the wider family – (Effect on wider family) | 2.8 | ‘When it happened it just shattered our family, my mom and stepfather were just devastated. It just left a big gaping hole in our family, which I think never quite, is going to go away.’ ^Avelin 2014^ | Kelley 2012, Downe 2013, Avelin 2014, O’Leary 2011 |
| For some parents QoL might be affected long term | 2.1 | Findings from quantitative studies only. | Gravensteen 2012, Sutan, 2012, Couto 2009 |
| Some couples experience conflicting emotional reactions to sexual relationships – (Sexual relationships) | 1.4 | ‘In the first period following the loss, sex was both hurtful and nice, hurtful because of guilt, I was not supposed to feel pleasure when she was dead, and still it was good because of the physical closeness.’ ^Dyregrov 2011^  [Several mothers] felt they had no "right" to enjoy anything when their child was dead: I could not feel pleasure with sex when my child was dead. In fact, I felt I should not feel pleasure over anything, as I was a horrible human being.’ ^Dyregrov 2011^ | Dyregrov 2011, Cacciatore 2008 |
